# *Aspergillus nidulans* transcription factor AtfA interacts with the MAPK SakA to regulate general stress responses, development and spore functions

**DOI:** 10.1111/j.1365-2958.2011.07581.x

**Published:** 2011-03-01

**Authors:** Fernando Lara-Rojas, Olivia Sánchez, Laura Kawasaki, Jesús Aguirre

**Affiliations:** Departamento de Biología Celular y Desarrollo, Instituto de Fisiología Celular, Universidad Nacional Autónoma de MéxicoApartado Postal 70-242, 04510, México, D.F., México

## Abstract

Fungi utilize a phosphorelay system coupled to a MAP kinase module for sensing and processing environmental signals. In *Aspergillus nidulans*, response regulator SskA transmits osmotic and oxidative stress signals to the stress MAPK (SAPK) SakA. Using a genetic approach together with GFP tagging and molecular bifluorescence we show that SakA and ATF/CREB transcription factor AtfA define a general stress-signalling pathway that plays differential roles in oxidative stress responses during growth and development. AtfA is permanently localized in the nucleus, while SakA accumulates in the nucleus in response to oxidative or osmotic stress signals or during normal spore development, where it physically interacts with AtfA. AtfA is required for expression of several genes, the conidial accumulation of SakA and the viability of conidia. Furthermore, SakA is active (phosphorylated) in asexual spores, remaining phosphorylated in dormant conidia and becoming dephosphorylated during germination. SakA phosphorylation in spores depends on certain (SskA) but not other (SrrA and NikA) components of the phosphorelay system. Constitutive phosphorylation of SakA induced by the fungicide fludioxonil prevents both, germ tube formation and nuclear division. Similarly, *Neurospora crassa* SakA orthologue OS-2 is phosphorylated in intact conidia and gets dephosphorylated during germination. We propose that SakA–AtfA interaction regulates gene expression during stress and conidiophore development and that SAPK phosphorylation is a conserved mechanism to regulate transitions between non-growing (spore) and growing (mycelia) states.

## Introduction

Growing evidence indicates that eukaryotic cells can modulate the production of reactive oxygen species (ROS) and use them as signalling molecules. However, little is known about how ROS are perceived or about how ROS can trigger different cell responses. After proposing that oxidative stress plays a key role in determining cell differentiation in fungi and other eukaryotes (Hansberg and Aguirre, 1990; Aguirre *et al*., 2005), we have been interested in the mechanisms involved in the production (Lara-Ortiz *et al*., 2003; Cano-Dominguez *et al*., 2008), perception (Kawasaki *et al*., 2002; Aguirre *et al*., 2006; Vargas-Perez *et al*., 2007) and elimination (Navarro *et al*., 1996; Kawasaki *et al*., 1997; Navarro and Aguirre, 1998; Kawasaki and Aguirre, 2001) of ROS in fungi.

Studies in unicellular yeast *Schizosaccharomyces pombe* have shown that this fungus utilizes a prokaryotic-type phosphorelay system coupled to a MAP kinase pathway to perceive and respond to high external ROS levels (Nguyen *et al*., 2000; Buck *et al*., 2001; Quinn *et al*., 2002; Ikner and Shiozaki, 2005). Fungal phosphorelays consist of one or several hybrid sensor kinases (HK), a histidine-containing phosphotransfer protein (HPt) and two canonical response regulators (RR). *S. pombe* contains three HKs, one Hpt protein called Mpr1 and the two RRs Mcs4 and Prr1. HKs Mak2 and Mak3 (Buck *et al*., 2001) transmit oxidative stress signals through Mcs4, to the stress MAPK (SAPK) Spc1/Sty1 (Nguyen *et al*., 2000; Buck *et al*., 2001). Once phosphorylated, Spc1/Sty1 phosphorylates Atf1 (Shiozaki and Russell, 1996; Wilkinson *et al*., 1996; Gaits *et al*., 1998), a transcription factor homologous to human ATF2 and Atf1 mediates most of the transcriptional responses regulated by Spc1/Sty1 (Chen *et al*., 2003).

Similar stress-activated protein kinase (SAPK) sensing pathways appear to operate in filamentous fungi such as the ascomycete *Aspergillus nidulans* (Virginia *et al*., 2000; Ochiai *et al*., 2001; Kawasaki *et al*., 2002; Zhang *et al*., 2002; Aguirre *et al*., 2005; 2006; Furukawa *et al*., 2005; Jones *et al*., 2007; Vargas-Perez *et al*., 2007). *A. nidulans* is a well-established genetic model that displays sophisticated patterns of multicellular asexual and sexual development. Asexual sporulation (conidiation) is triggered by environmental signals such as exposure to air (Clutterbuck, 1969; Timberlake and Clutterbuck, 1994; Adams *et al*., 1998), starvation for nutrients (Skromne *et al*., 1995) and the presence of self-generated chemical signals (Lee and Adams, 1996; Seo *et al*., 2003; Soid-Raggi *et al*., 2006; Marquez-Fernandez *et al*., 2007; Tsitsigiannis and Keller, 2007). In an air interphase, conidiation initiates with the formation of a cell compartment called the foot cell, which develops a conidiophore stalk that grows by apical extension towards the air. At a fixed length, the tip of the conidiophore stalk swells to form a multinucleated vesicle, from which multiple uninucleated buds, called metulae, are produced. The metulae bud to produce a second tier of uninucleated sporogenic cells called phialides. The asexual spores or conidia are produced by repeated mitotic divisions of the phialide nucleus in such a way that one daughter nucleus enters the developing spore and the other one is retained in the phialide to undergo division (Clutterbuck, 1969; Timberlake and Clutterbuck, 1994). In contrast, the spore nucleus becomes arrested at the G1 phase of the cell cycle (Bergen and Morris, 1983). As other types of spores, fungal conidia are dispersal structures characterized by being long-lived dormant cells, and by their resistance to different environmental insults. In the presence of nutrients, *A. nidulans* conidia germinate undergoing an initial period of isotropic growth, followed by the formation of an elongating germ tube. Coupled with these morphological changes, spores resume metabolism and re-enter the nuclear division cycle (Harris, 1999). Asexual development is often followed by sexual differentiation, which involves the formation of dark, round multicellular fruiting bodies called cleistothecia, inside of which the meiotic spores (ascospores) are formed.

Compared with *S. pombe* and other unicellular fungi, filamentous fungi show additional mechanisms to handle ROS, such as the presence of a larger number of antioxidant enzymes (Kawasaki and Aguirre, 2001; Aguirre *et al*., 2005) and secondary metabolites (Yu and Keller, 2005; Thines *et al*., 2006; Marquez-Fernandez *et al*., 2007), some already tested for antioxidant function (Lee *et al*., 2005). Furthermore, filamentous fungi have enzymes like the NADPH oxidases, which regulate sexual development through ROS production (Lara-Ortiz *et al*., 2003; Aguirre *et al*., 2005; Aguirre and Lambeth, 2010). Therefore, filamentous fungi provide an opportunity to study interactions between ROS production, perception and detoxification, and how these processes relate to cell differentiation (Aguirre *et al*., 2005) and secondary metabolism and pathogenesis (Aguirre *et al*., 2006). In this context, we characterized *A. nidulans* stress activated MAP kinase SakA (Kawasaki *et al*., 2002), also called HogA (Han and Prade, 2002). SakA is able to replace Spc1 functions in *S. pombe* and is activated by osmotic and oxidative stress signals in *A. nidulans* (Kawasaki *et al*., 2002), a response conserved in other filamentous fungi (Segmuller *et al*., 2007; Eaton *et al*., 2008). The activation of SakA by osmotic and oxidative stress is mediated by response regulator SskA (Furukawa *et al*., 2005). In addition, SakA is involved in repression of sexual development and is required for viability of the asexual spores (Kawasaki *et al*., 2002).

We identified an orthologue of *S. pombe* Atf1 and proposed that it could function as downstream component of the SakA pathway (Aguirre *et al*., 2005; 2006;). Recently, several Atf1 orthologues have been studied in filamentous fungi. *Claviceps purpurea cptf1*, identified as a gene expressed *in planta*, is involved in virulence and regulation of the *cpcat1* gene, encoding a secreted catalase, and possibly other catalase genes (Nathues *et al*., 2004). *Aspergillus oryzae atfB*, predicting a small protein more distantly related to Atf1, was identified as a gene expressed during growth on solid-state culture. Deletion of *A. oryzae atfB* gene resulted in a decrease in mRNA level of several genes normally upregulated during growth on solid medium, including the catalase gene *catA*. In addition, the *atfB* mutant presented a slight decrease in conidiation and produced conidia that germinated normally but were sensitive to high (> 250 mM) H_2_O_2_ concentrations (Sakamoto *et al*., 2008). Results published while this work was in preparation show that *A. nidulans sakA* and *atfA* mutants share some phenotypes and regulate several genes in common under oxidative, osmotic or specific fungicide treatments (Hagiwara *et al*., 2008; 2009; Balazs *et al*., 2010). However, direct evidence showing that AtfA functions downstream and interacts with SakA under stress conditions has been missing. Furthermore, very little is known about SakA functions under other stress conditions or during development.

Here we demonstrate that *A. nidulans atfA* gene encodes a nuclear protein that interacts with SakA in response to stress and differentially regulates the antioxidant response in asexual spores versus mycelia. We show that AtfA determines SakA protein levels in conidia but not in mycelia and in doing so regulates the viability of the spores. Furthermore, we show that SakA interacts with AtfA during conidiophore development and is active (phosphorylated) in dormant asexual spores and that SakA phosphorylation levels regulate the transition between spore dormancy, germination and nuclear division. We report a similar behaviour for the MAPK OS-2 in conidia from the distantly related fungus *Neurospora crassa*, and propose that SAPK phosphorylation in spores is a general mechanism to regulate the transition between non-growing and growing states in fungi and other eukaryotes.

## Results

### The *atfA* gene encodes a putative bZIP transcription factor of the ATF/CREB family

We proposed that filamentous fungi use a stress MAPK pathway similar to the one present in *S*. *pombe*, to transduce environmental stress signals (Kawasaki *et al*., 2002; Aguirre *et al*., 2005; 2006;), in which transcription factor Atf1 mediates multiple responses to stress (Wilkinson *et al*., 1996; Gaits *et al*., 1998; Chen *et al*., 2003). To identify Atf1 orthologues in *A. nidulans*, we searched a public cDNA database (Prade *et al*., 2001), using Atf1 protein sequence as query. A partial cDNA was recognized and used to identify cosmid W16G04, which was then used to obtain the genomic sequence deposited earlier at GenBank (*atfA* gene; Accession No. AY166595). Based on this, as well as in cDNA sequencing, *atfA* encodes a protein of 485 amino acids, identical to protein AN2911.3 derived from the *A. nidulans* genomic sequence (Galagan *et al*., 2005), published subsequently. AtfA contains a bZIP domain very similar to the one present in *S. pombe* Atf1 and orthologues from other filamentous fungi (Fig. S1). This is in agreement with Hagiwara *et al*. (2008) and suggests that AtfA is a transcription factor of the ATF/CREB (activating transcription factor/cAMP-responsive element-binding protein) family.

### *sakA* is epistatic to *atfA*; the SakA–AtfA pathway regulates different antioxidant responses in spores and mycelia

**Fig. 1:**
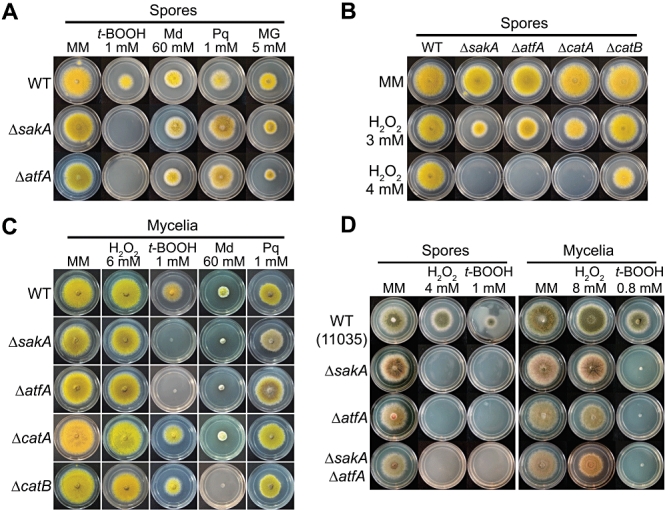
*sakA* is epistatic to *atfA*; the SakA–AtfA pathway regulates different antioxidant responses in spores versus mycelia. A. Conidia (1 × 10^4^) from strains CLK43 (wild type; WT), TOL1 (Δ*sakA*) and TFLΔatfA-02 (Δ*atfA*) were inoculated on supplemented MM plates containing *t*-butylhydroperoxide (*t*-BOOH), menadione (Md), paraquat (Pq) or methylglyoxal (MG), at the indicated concentrations, and incubated at 37°C for 4 days. B. Conidia from strains CLK43 (WT), TOL1 (Δ*sakA*), TFLΔatfA-02 (Δ*atfA*), TRN1 (Δ*catA*) and TLK12 (Δ*catB*) were inoculated as in (A) on plates containing 3 or 4 mM H_2_O_2_. C. Mycelial plugs cut from the growing edge of 5-day colonies from strains CLK43 (WT), TOL1 (Δ*sakA*), TFLΔatfA-02 (Δ*atfA*), TRN1 (Δ*catA*) and TLK12 (Δ*catB*) were transferred to plates containing the indicated compounds and incubated at 37°C during 4 days. D. Conidia (1 × 10^4^) and mycelial plugs from strains 11035 (WT), TFLΔsakA-03 (Δ*sakA*), TFLΔatfA-04 (Δ*atfA*) and TFL4 (Δ*sakA ΔatfA*) were inoculated on plates containing the indicated concentrations of H_2_O_2_ and *t*-butylhydroperoxide (*t*-BOOH) and incubated for 4 days at 37°C.See Table 2 for full strain genotypes.

To evaluate *atfA* functions and possible connections to the SakA MAPK pathway, we first generated strains carrying complete deletions in either gene, as confirmed by Southern blot analysis (Figs S2 and S3A and B). Δ*atfA* and Δ*sakA* mutants were indistinguishable from the wild-type strain under high-temperature (42°C) or high-osmolarity (1 M NaCl or 1.2 M sorbitol) stress conditions (not shown). To test the mutant response to different types of oxidative stress, we incubated Δ*atfA* and Δ*sakA* strains in the presence of the redox-cycling compounds menadione and paraquat, the glutathione-depleting compound methylglyoxal and inorganic (H_2_O_2_) as well as organic (*t*-butylhydroperoxide; *t*-BOOH) peroxides. As shown in Fig. 1A, wild-type, Δ*atfA* and Δ*sakA* strains were similarly resistant to menadione and paraquat. In contrast, Δ*atfA* and Δ*sakA* mutants were hypersensitive to both *t*-butylhydroperoxide (Fig. 1A) and hydrogen peroxide (Fig. 1B), showing a slight sensitivity to methylglyoxal (Fig. 1A). Notably, Δ*atfA* and Δ*sakA* mutants were as sensitive to H_2_O_2_ as the Δ*catA* mutant, which lacks the spore-specific catalase CatA (Navarro *et al*., 1996; Navarro and Aguirre, 1998). On the contrary, a mutant lacking the mycelial inducible catalase CatB (Kawasaki *et al*., 1997) showed a H_2_O_2_ resistance only slightly lower than the wild type (Fig. 1B). This and the fact that conidia from *sakA* null mutants show decreased CatA activity (Kawasaki *et al*., 2002) suggested that under these conditions, mutant sensitivity to H_2_O_2_ could reflect low CatA activity in conidia. To explore this, we carried out similar oxidative stress plate assays but using mycelial plugs instead of conidia. As shown in Fig. 1C, mycelia from Δ*atfA* and Δ*sakA* mutants was resistant up to 6 mM H_2_O_2_ but resulted hypersensitive to *t*-BOOH. Notably, under these conditions, mycelia from Δ*catA*, Δ*catB* and wild-type strains showed similar resistance to H_2_O_2_ and *t*-BOOH. Compared with the wild-type, Δ*atfA*, Δ*sakA* and Δ*catB* mutants were somewhat more sensitive to menadione. While all strains presented similar growth in paraquat, a brownish pigmentation and decreased conidiation was observed in Δ*atfA* and Δ*sakA* mutants (Fig. 1C). Results published during the course of this work show that conidia from Δ*atfA* (Δ*sakA* was not analysed) mutants were sensitive to 50 mM H_2_O_2_ (*t*-BOOH was not tested), while Δ*atfA* mycelia was resistant to 1.2 mM *t*-BOOH (Hagiwara *et al*., 2008). In agreement with our results, a more recent report shows that Δ*atfA* mycelium is indeed sensitive to *t*-BOOH (Balazs *et al*., 2010).

We tried to generate Δ*sakA*Δ*atfA* double mutants to gather additional evidence of genetic interaction. However, in repeated attempts, we were unable to isolate hybrid cleistothecia from sexual crosses between Δ*sakA* and Δ*atfA* strains. Therefore, we carried out the sequential deletion of both genes (Fig. S3) and analysed the phenotype of resulting single and double mutants, despite that these strains carried different auxotrophic markers. Under this genetic background, conidiation was decreased more in the Δ*sakA* than in the Δ*atfA* strains. Nevertheless, spores and mycelia from Δ*sakA*, Δ*atfA* and Δ*sakA*Δ*atfA* mutants presented similar patterns of sensitivity to oxidative (Fig. 1D) and osmotic stress, and showed derepression of sexual development (not shown). Moreover, spores from double and single mutants showed the same sensitivity at lower concentrations of H_2_O_2_ and *t*-BOOH (Fig. S4). Notably, the mycelia from Δ*sakA*, Δ*atfA* and Δ*sakA*Δ*atfA* mutants were highly resistant to H_2_O_2_ but hypersensitive to *t*-BOOH (Fig. 1D). These results further indicate that SakA and AtfA are components of the same signal transduction pathway. In addition, they show that the oxidative stress responses of conidia and mycelia are different and that under the conditions tested, *sakA* and *atfA* play a minor role in mycelial resistance to H_2_O_2_. Nevertheless, both genes are essential for spore and mycelial resistance to *t*-BOOH.

### AtfA and SakA regulate catalase expression during development and in response to different types of stress

**Fig. 2:**
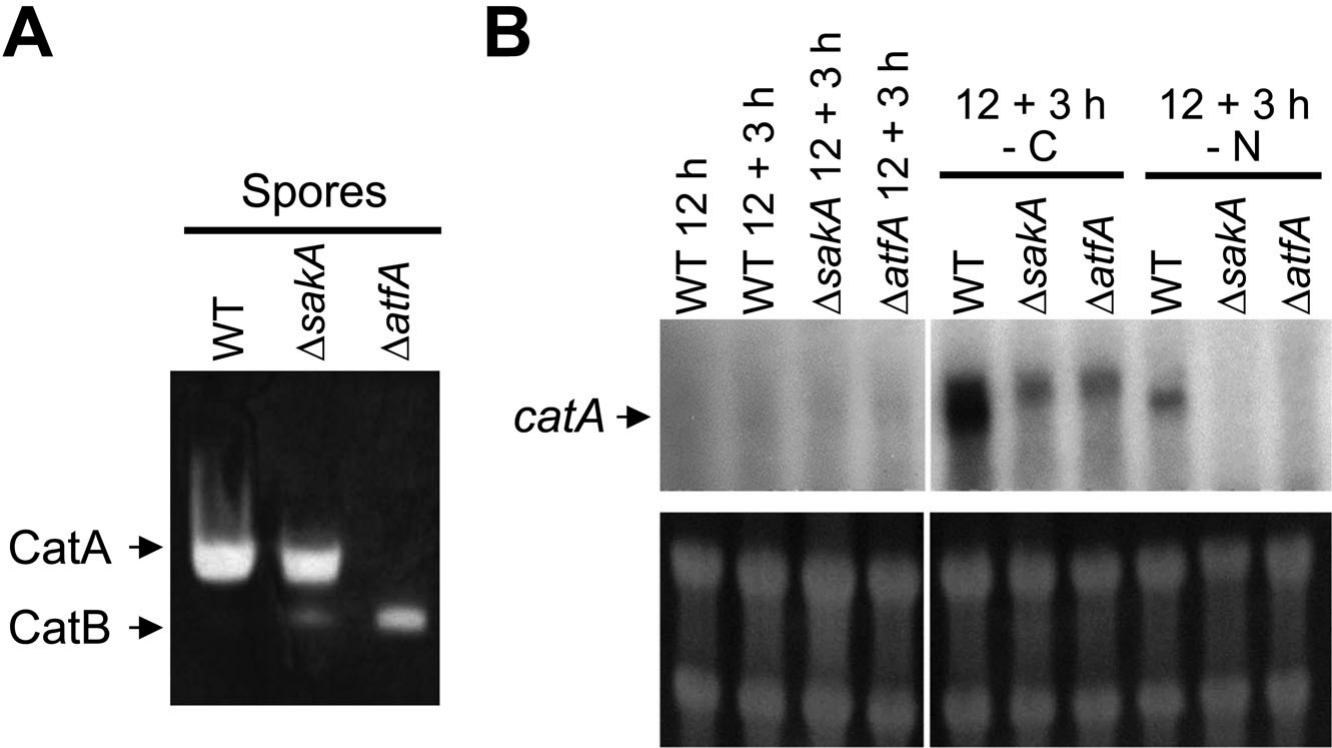
Conidia from Δ*atfA* mutant lack CatA catalase activity; *sakA* and *atfA* genes are required for *catA* mRNA accumulation in response to nutrient starvation. A. Thirty micrograms of protein extracts prepared from conidiospores of strains CLK43 (WT), TOL1 (Δ*sakA*), TFLΔatfA-02 (Δ*atfA*) were separated on native polyacrylamide gels to determine catalase activity as described previously (Navarro *et al*., 1996). B. Mycelia from the same strains was grown for 12 h and shifted to the indicated nutrient stress condition for 3 h. As reported before, changes in *catA* mRNA size are observed under determined stress conditions (Navarro and Aguirre, 1998). Samples were collected to extract total RNA and used for Northern hybridization using a *catA*-specific probe. Bottom panel shows ribosomal RNA as loading controls.

The H_2_O_2_ sensitivity observed in Δ*atfA* and Δ*sakA* mutants suggested that AtfA and SakA could play a role in the regulation of catalase genes *catA* and *catB*, as well as in the regulation of other genes specifically involved in the response to oxidative stress caused by *t*-BOOH. To determine if sensitivity to H_2_O_2_ was related to catalase gene expression, we first examined catalase activity in conidial samples from wild-type, Δ*atfA* and Δ*sakA* strains. Consistent with previous findings (Kawasaki *et al*., 2002; Vargas-Perez *et al*., 2007), CatA activity levels in Δ*sakA* conidia were lower than in wild-type conidia. This situation was more severe in the Δ*atfA* mutant, as no CatA activity was detected in Δ*atfA* conidia (Fig. 2A), a result consistent with the absence of *catA* mRNA in Δ*atfA* conidia (Hagiwara *et al*., 2008). However, as *catA* mRNA accumulates in response to different types of stress, some of which induce conidiation (Navarro and Aguirre, 1998), we examined *catA* mRNA levels under nutrient starvation conditions. As shown in Fig. 2B, *catA* mRNA did not accumulate when mycelia grown for 12 h was transferred to regular media for another 3 h. In contrast, *catA* mRNA accumulated in response to nutrient starvation and the lack of *sakA* or *atfA* had negative effects on *catA* mRNA levels. While *sakA* and *atfA* were partially required for high *catA* mRNA levels in response to carbon starvation, either gene was essential for increased *catA* mRNA levels under nitrogen starvation. These results indicate that AtfA and SakA function in the same pathway to regulate *catA* expression during development and in response to stress, and show that the SakA pathway is involved in transducing carbon and nitrogen starvation signals.

We also asked whether AtfA or SakA were involved in induction of the mycelial catalase CatB. The induction of CatB observed during the stationary phase of growth or during paraquat treatment (Kawasaki *et al*., 1997) was largely unaffected by the inactivation of *sakA* or *atfA*, while CatB induction by H_2_O_2_ was slightly decreased in Δ*sakA* and Δ*atfA* strains (not shown). Under the same conditions, the levels of activity of the Cu/Zn superoxide dismutase SodA (Oberegger *et al*., 2000) were not affected by the lack of *atfA* or *sakA* (not shown). These results show that SakA and AtfA play a critical role in *catA* regulation and suggest that both genes are needed for full induction of CatB in response to H_2_O_2_, a result consistent with a recent report (Balazs *et al*., 2010).

### Lack of *atfA*, as the lack of *sakA*, results in derepressed sexual development

**Fig. 3:**
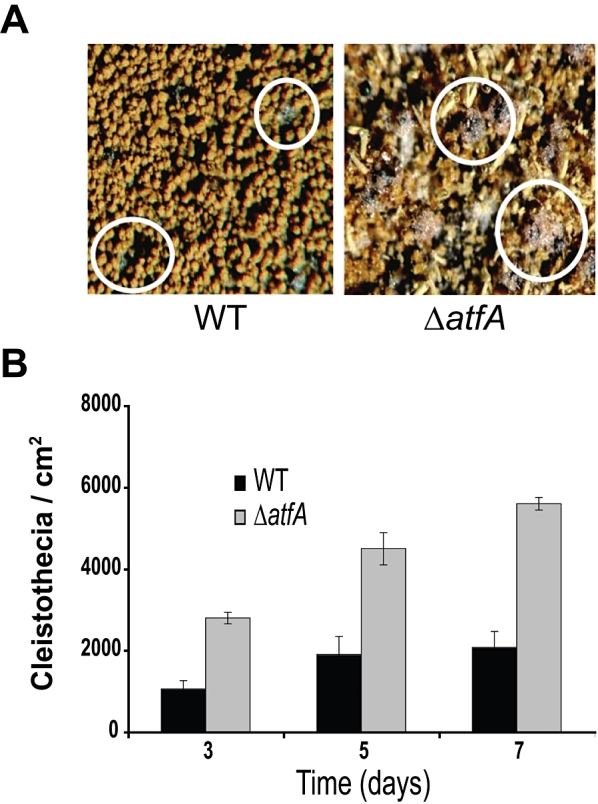
The deletion of *atfA* causes derepression of sexual development. A. CLK43 (WT) and TFLΔatfA-02 (Δ*atfA*) strains were induced to undergo sexual development in confluent plates as previously reported (Kawasaki *et al*., 2002). Pictures were taken from confluent cultures after 10 days of induction. Under these conditions the Δ*atfA* mutant produced more Hülle cells and cleistothecia (black spheres; some in circles) than the WT. B. Strains CLK43 (WT) or TFLΔatfA-02 (Δ*atfA*) were induced to undergo sexual development as (A) and samples were taken every 24 h (see *Experimental procedures*). The total number of cleistothecia per fixed area was counted under a dissection microscope and used to calculate cleistothecia cm^−2^ as reported (Kawasaki *et al*., 2002). Data are mean values from three independent experiments; bars indicate standard deviation.

Since the inactivation of *sakA* causes a derepression of sexual development (Kawasaki *et al*., 2002; Lara-Ortiz *et al*., 2003), we followed the development of sexual fruiting bodies (cleistothecia) in the Δ*atfA* mutant. As shown in Fig. 3A and B, the elimination of *atfA* also resulted in derepressed sexual development and higher numbers of cleistothecia, suggesting that SakA and AtfA repress sexual development through the same pathway.

### AtfA is localized in the nucleus in the absence of stress, while SakA accumulates in the nuclei and interacts with AtfA in response to oxidative stress

**Fig. 4:**
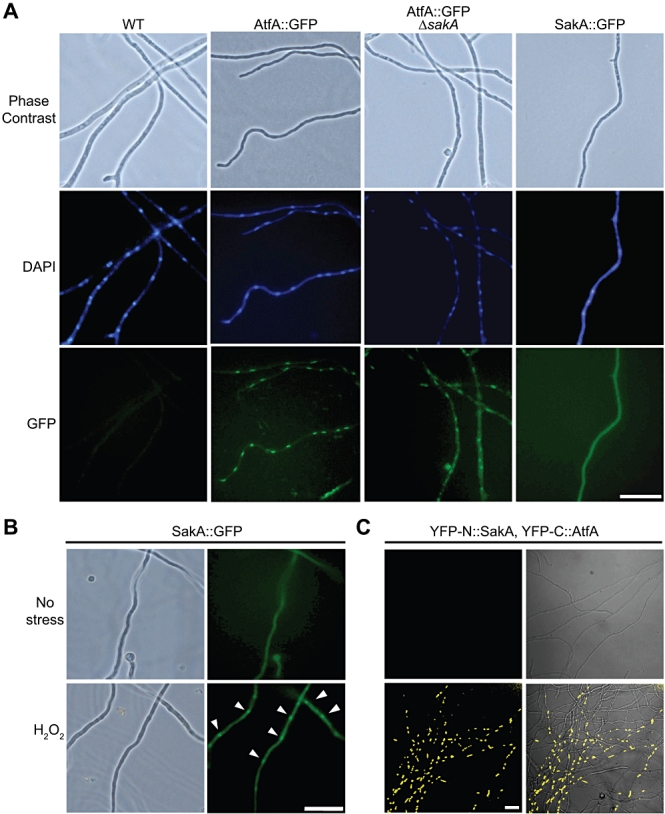
AtfA::GFP shows nuclear localization independently of *sakA*; SakA accumulates in the nucleus in response to hydrogen peroxide stress, where it interacts with transcription factor AtfA. A. Conidia from strains CLK43 (WT), TFL3 (AtfA::GFP), CFL9 (Δ*sakA*; AtfA::GFP) and TFL6 (SakA::GFP) were inoculated on coverslips submerged in liquid supplemented minimal medium, incubated for 12 h at 37°C and then fixed and stained with DAPI. B. Conidia from strain TFL6 were inoculated as in (A) and then treated with 30 mM H_2_O_2_ for 30 min. Arrowheads indicate SakA::GFP localization after stress treatment. Pictures were taken using Epifluorescence with a microscope NIKON Eclipse E600. C. BiFC analysis of SakA and AtfA. Strain TFL7 expressing YFP-N::SakA and YFP-C::AtfA was grown as in (B), transferred to minimal medium with 100 mM threonine for 3 h to induce the *alcA* promoter, and exposed to 30 mM H_2_O_2_ for 30 min. Yellow fluorescence was detected using a confocal microscope Olympus FV1000.Bar = 20 µm.

Our results above suggest a model in which the MAPK SakA regulates putative transcription factor AtfA, in response to different stress signals. To examine AtfA and SakA localization we generated strains TFL3 and TFL6, which carry *atfA::gfp* or *sakA::gfp* alleles replacing the wild-type genes respectively (confirmed by Southern blot analysis; not shown). As shown in Fig. S5, conidia from strains TFL3 and TFL6 were not sensitive to H_2_O_2_, indicating that GFP tagging did not interfere with AtfA or SakA functions. In addition, the *atfA::gfp* allele was introduced into a Δ*sakA* genetic background by sexual crosses, to generate strain CFL9, and the presence of both mutant alleles was confirmed by Southern analysis (not shown). When these strains were used to detect GFP localization, we found AtfA::GFP in the nucleus even in the absence of any experimentally induced stress. Furthermore, such nuclear localization was largely unaffected by the absence of the MAPK SakA (Fig. 4A). In contrast, SakA::GFP fluorescence was not restricted to DAPI-stained nuclei, showing a more uniform distribution in hyphae (Fig. 4A, right panels). These results supported AtfA function as transcription factor and suggested that to regulate AtfA, SakA would need to be translocated to the nucleus. Indeed, treatment with H_2_O_2_ induced a re-distribution of SakA::GFP fluorescence consistent with the nuclear localization of SakA in response to oxidative stress (Fig. 4B).

The physical interaction between SakA and AtfA in response to oxidative stress was evaluated *in vivo* by using the bimolecular fluorescence complementation assay (BiFC), as reported by Takeshita *et al*. (2008). The N-terminal half of the yellow fluorescent protein (YFP-N) was fused to the N-terminus of SakA and the C-terminal half of YFP (YFP-C) was fused to N-terminus of AtfA, both fusions under the control of the *alcA* gene promoter. As shown in Fig. 4C, in the absence of H_2_O_2_ the coexpression of YFP-N::SakA and YFP-C::AtfA did not produce fluorescence. In contrast, in the presence of H_2_O_2_ YFP signal was reconstituted and localized in nuclei. Similarly, SakA and AtfA interaction was detected under osmotic stress conditions (not shown).

### Asexual spores from Δ*atfA* mutants show decreased viability and lack SakA

**Fig. 5:**
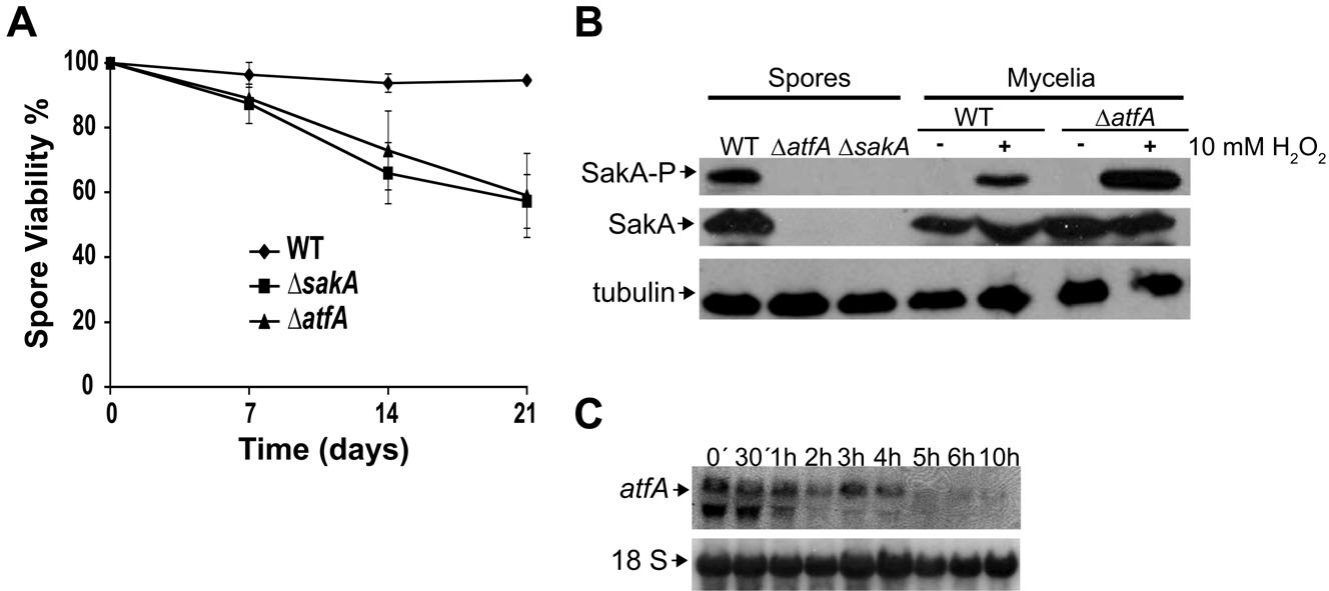
Asexual spores from Δ*sakA* and Δ*atfA* mutants show decreasing viability and lack SakA. A. Conidia from strains CLK43 (WT), TOL1 (Δ*sakA*) and TFLΔatfA-02 (Δ*atfA*) were collected from 5-day plates, counted and plated immediately (time 0) or maintained in water at 4°C for up to 21 days. At indicated time points, aliquots were diluted and used to inoculate supplemented MM plates, which were incubated at 37°C for 2 days and resulting colonies were counted. Data are mean values from three independent experiments; bars indicate standard deviation. B. Freshly collected conidia or mycelial samples from strains indicated in (A) were frozen with liquid nitrogen and used to prepare total protein extracts, followed by immunoblotting (50 µg), with anti-Hog1 and anti-Phospho-p38 or anti-tubulin antibodies as reported (Kawasaki *et al*., 2002). C. Total RNA isolated from intact or germinated conidia from strain CLK43 (WT) was used for northern hybridization with probes against *atfA* or the 18S ribosomal RNA gene as a loading control.

**Fig. 6:**
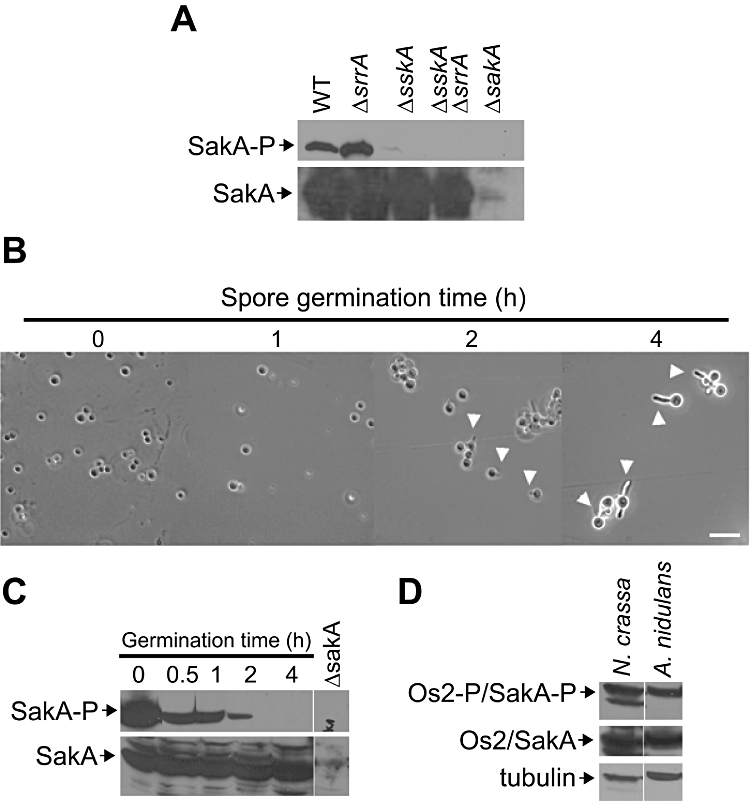
*A. nidulans* SakA and *N. crassa* OS-2 SAPKS are phosphorylated in intact conidia; SakA dephosphorylation occurs during germination. A. Conidia from strains CLK43 (WT), COSΔsrrA03 (Δ*srrA*), COSΔsskA02 (Δ*sskA*), COSΔsrrA/ΔsskA02 (Δ*srrA*Δ*sskA*) and TOL1 (Δ*sakA*) were collected, immediately frozen with liquid nitrogen and processed for Western blot immunodetection of phosphorylated (SakA-P). Δ*sakA* strain TOL1 was included as negative control and a total SakA blot is included as protein loading control. B and C. (B) Conidia from wild-type strain CLK43 were used to inoculate MM and incubated at 37°C for up to 4 h. At indicated time points samples were collected, examined by light microscopy (arrowheads indicate some of the conidial germ tubes) and processed as in (A) for immunoblotting, to detect phosphorylated (SakA-P) and total SakA (C). Bar = 10 µm. D. Conidia from *N. crassa* or *A. nidulans* were processed for immunoblotting as in (A) to detect phosphorylated and non-phosphorylated levels of MAPK OS-2 (*N. crassa*) or SakA (*A. nidulans*). An anti-tubulin antibody was used as additional sample loading reference.See Table 2 for full strain genotypes.

The fact that Δ*sakA* conidia lose their viability after prolonged storage (Kawasaki *et al*., 2002) prompted us to compare conidial viability in Δ*sakA* and Δ*atfA* mutants. Results in Fig. 5A show that both mutants produced conidia that gradually lost their viability with similar kinetics, while wild-type conidia retained full viability under the same conditions. Although Hawigara *et al*. have reported comparable results for Δ*atfA* conidia, it is not clear why under similar conditions they observed a complete loss of viability after 10 days (Hagiwara *et al*., 2008). When we examined SakA levels in wild-type and mutant conidia, we observed high levels of SakA in intact conidia from the wild-type strain, while SakA was almost undetectable in conidia from the Δ*atfA* mutant (Fig. 5B, left lanes). In contrast, AtfA was not required to maintain SakA levels or for SakA phosphorylation in response to H_2_O_2_, in mycelial samples (Fig. 5B, right lanes). Notably, our results also show that SakA is phosphorylated (active) in wild-type conidia (Fig. 5B). When *atfA* mRNA was examined in intact conidia and during germination we detected two transcripts with the probe used. However, only the larger transcript was missing in Δ*atfA* mutants (not shown) and therefore it corresponds to *atfA*. Both transcripts were accumulated in intact conidia and gradually decreased during spore germination. The *atfA* transcript showed lower levels after 5 h of germination (Fig. 5C), a time when all spores have germinated (see Fig. 6B). Our results suggest a connection between SakA phosphorylation and spore viability, show that AtfA is required for SakA accumulation in asexual spores but not in mycelia, and that SakA becomes phosphorylated during normal asexual development.

### *A. nidulans* SakA and *N. crassa* OS-2 SAPKs are phosphorylated in asexual spores; SakA phosphorylation regulates spore dormancy and germination

As indicated, SakA is phosphorylated in response to osmotic and oxidative stress signals (Kawasaki *et al*., 2002) in a process that requires response regulator SskA (Furukawa *et al*., 2005; Vargas-Perez *et al*., 2007). Having found SakA phosphorylated in dormant spores, we asked if this process was also dependent on SskA. Indeed, SakA phosphorylation in conidia required SskA but was independent of response regulator SrrA (Fig. 6A). Although histidine kinase NikA mediates SskA activation in response to the fungicide fludioxonil (Vargas-Perez *et al*., 2007), it was dispensable for SakA activation in spores (not shown). SakA remained phosphorylated in wild-type conidia even after prolonged incubation (up to 7 days) in water at 4°C (data not shown), a condition in which *A. nidulans* conidia do not germinate and remain viable. In contrast, SakA phosphorylation decreased during spore germination in minimal medium with glucose and was completely lost after 4 h (Fig. 6C), a time at which virtually all conidia had produced a germ tube (Fig. 6B). This and the fact that conidia lacking SakA (Δ*sakA* and Δ*atfA* mutants; Fig. 5A) loose viability over time indicate that SakA phosphorylation is essential to maintain spore dormancy and viability. According to this, one would expect that members of this family of stress MAPKs would play similar roles in other fungi. We examined the phosphorylation levels of the SakA orthologue OS-2 in conidia from *N. crassa* and as observed for SakA, OS-2 was phosphorylated in intact conidia (Fig. 6D) and became dephosphorylated during spore germination (not shown).

**Fig. 7:**
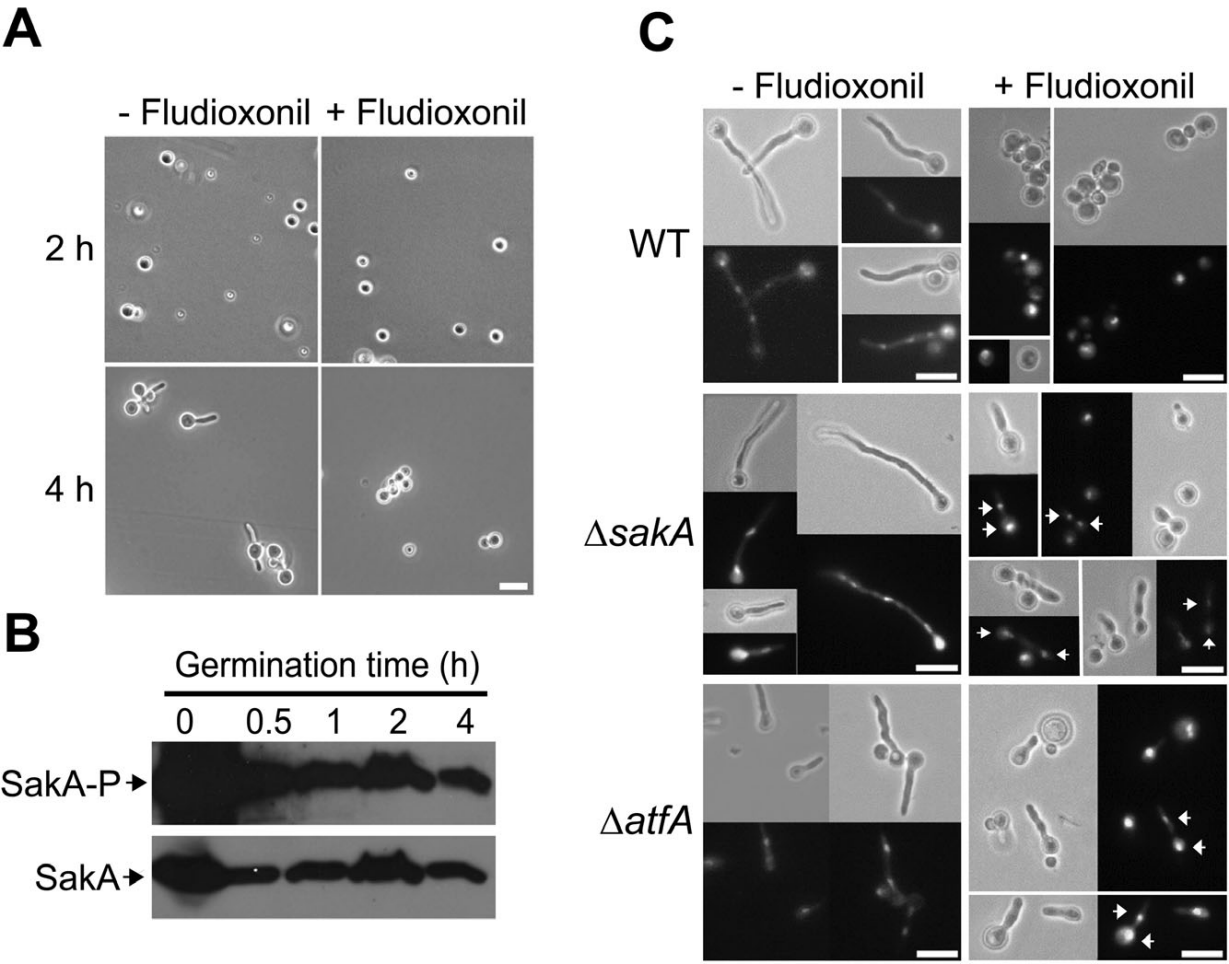
Fungicide fludioxonil prevents conidia germination, SakA dephosphorylation and nuclear division in a SakA-dependent manner. A. Conidia from wild-type strain CLK43 were inoculated in MM in the absence (−) or the presence (+) of fludioxonil (2 µg ml^−1^) for up to 4 h. Samples were taken at indicated time points and examined by light microscopy. B. Fludioxonil-treated samples from (A) were collected, frozen with liquid nitrogen and processed for immunodetection as in Fig. 6. C. Conidia from strains CLK43 (WT), TOL1 (Δ*sakA*) and TFLΔatfA-02 (Δ*atfA*) were used to inoculate 50 ml of cultures plus and minus fludioxonil, incubated for 6 h and fixed. Nuclei were stained with DAPI and examined by phase-contrast (grey panels) and fluorescence microscopy (dark panels). Arrowheads indicate the presence of two nuclei in a single germinated spore.Bars = 10 µm.

To provide additional support to the idea that phosphorylated SakA maintains spore dormancy and/or prevents germination, we used the fungicide fludioxonil, which is known to result in constitutive phosphorylation of SakA orthologues (Zhang *et al*., 2002; Kojima *et al*., 2004). Results show that in the presence of fludioxonil wild-type conidia failed to produce germ tubes (Fig. 7A) and that under these conditions SakA remained phosphorylated (Fig. 7B). To test if SakA mediated this fludioxonil effect on spore germination and if such effect was related to nuclear division, we incubated spores (uninucleated) from wild-type, Δ*sakA* and Δ*atfA* strains with and without fludioxonil for up to 6 h. In the absence of the fungicide, conidia from all three strains formed germ tubes and two nuclei were detected by DAPI staining. In the presence of fludioxonil, wild-type conidia became swollen and none formed a germ tube, while DAPI staining of these spores produced a single, diffuse fluorescent signal that suggested the absence of nuclear division. In contrast, a high proportion of Δ*sakA* and Δ*atfA* conidia was able to develop a germ tube in the presence of the fungicide and showed two nuclei (Fig. 7C). Moreover, on solid medium containing fludioxonil, Δ*sakA* and Δ*atfA* conidia were able to form small colonies composed of short and aberrant hyphae (Fig. S6), while again wild-type conidia did swell but failed to generate any germ tube. As indicated, fludioxonil toxicity in *A. nidulans* is also mediated by the response regulator SrrA and full resistance is possible only if histidine kinase NikA or both response regulators (SrrA and SskA) are inactivated (Vargas-Perez *et al*., 2007).

### SakA is partially localized in the nucleus in spores and interacts with AtfA during conidiophore development

**Fig. 8:**
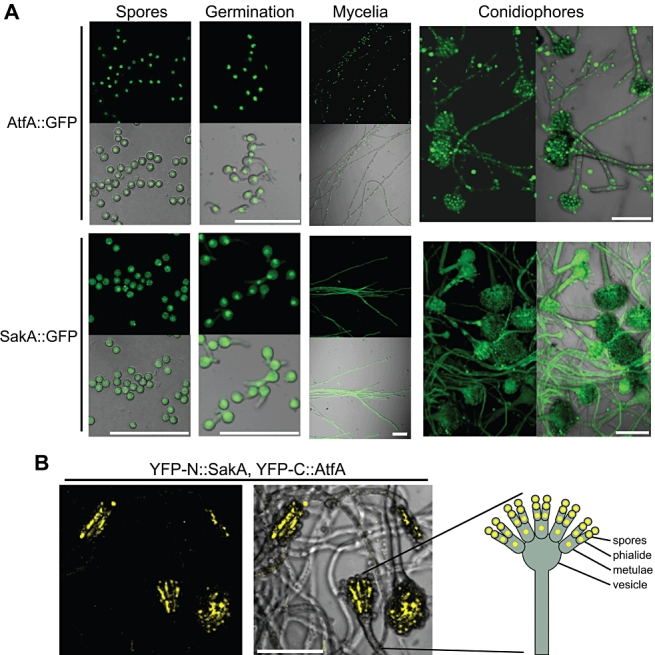
SakA and AtfA localization during growth and development; SakA and AtfA interact during conidiophore development. A. Spores from strains TFL3 (AtfA::GFP) and TFL6 (SakA::GFP) were inoculated on supplemented MM plates and observed before germination (Spores), 4 h after germination (Germination), after 24 h of growth (Mycelia) and after 48 h of induction of conidiation (Conidiophores). B. Strain TFL7, expressing YFP-N::SakA and YFP-C::AtfA, was inoculated on a plate containing MM and 100 mM threonine, to induce the *alcA* promoter, and observed after 48 h. A schematic representation of a conidiophore indicates cell types showing YFP fluorescence. Pictures show *Z* stacks of confocal images obtained with the confocal microscope Olympus FV1000.Bars = 30 µm.

Our results indicate that SakA is phosphorylated and accumulated in the nucleus not only in response to oxidative stress but also during normal spore development. To provide further support to this, we followed SakA::GFP and AtfA::GFP localization during development. AtfA::GFP showed nuclear localization in intact and germinating spores and throughout mycelial growth and conidiophore development (Fig. 8A, top panels). SakA::GFP showed discrete as well as evenly distributed signals in intact and germinating spores, while fluorescence was homogeneously distributed in growing hyphae (Fig. 8A, bottom panels). In contrast to AtfA::GFP, SakA::GFP signal showed a more uniform localization within the different conidiophore cell types, as well as in some patches (Fig. 8A, bottom panels). SakA localization results are consistent with its phosphorylation in conidia and suggest that SakA becomes partially phosphorylated during conidiophore development. Taking advantage of the fact that the *alcA* promoter is derepressed during conidiophore formation (Marhoul, 1996), we also used BiFC to evaluate the physical interaction of SakA and AtfA during conidiophore development. Although fluorescence was not detected in every cell, it was possible to clearly detect YFP signal in different conidiophore cell types, including metulae, phialides and developing spores (Fig. 8B). These results suggest that these cell types are under physiological stress and that the SakA–AtfA interaction during conidiophore development is critical in regulating gene expression and the developmental stages that culminate with the formation of viable dormant spores.

In summary, we have obtained conclusive evidence showing that AtfA functions downstream of the MAPK SakA. AtfA is constitutively localized in the nucleus independently of SakA while SakA accumulates in the nucleus in response to oxidative and osmotic stress, showing physical *in vivo* interaction with AtfA. SakA is also involved in transducing nutrient starvation signals and although the SakA–AtfA module plays critical roles in the antioxidant response in both, conidia and mycelia, these roles are clearly different. Furthermore, we found that SakA is in its active form (phosphorylated) in asexual spores and gets dephosphorylated during the germination process that includes germ tube formation and nuclear division and this also occurs in the distantly related fungus *N. crassa*, suggesting that this is conserved in spores from many if not all fungi. SakA phosphorylation in spores depends on certain (SskA) but not other (SrrA and NikA) components of the *A. nidulans* phosphorelay system and its maintenance prevents both, germ tube formation and nuclear division. SakA accumulation in spores but not in mycelia depends on AtfA itself. As seen through *in vivo* interaction with AtfA, the developmental activation of SakA occurs during conidiophore development. These results, not published before in any fungi, contribute to understand the mechanisms that regulate spore development, spore viability and germination, all processes with numerous implications in fungal biology.

## Discussion

### AtfA is a bZIP transcription factor involved in stress signalling

Our results support the role of AtfA as a transcription factor in *A. nidulans* showing that AtfA is constitutively localized in the nucleus and it is needed for full expression of catalase genes *catA* and *catB*, unidentified genes involved in *t*-BOOH detoxification, and very importantly, it is required for the presence of SakA in asexual spores. During the preparation/revision of this article, Hagiwara *et al*. reported that AtfA is involved in the expression of *catA* (Hagiwara *et al*., 2008) and that Δ*atfA* and Δ*sakA* mutants are similarly affected in the expression of genes induced by osmotic stress or fludioxonil treatment (Hagiwara *et al*., 2009), while Balazs *et al*. reported that heterologous expression of *atfA* in a *S. pombe Atf1* mutant can restore osmotic stress tolerance and that AtfA regulates *catB* and other genes in *A. nidulans* (Balazs *et al*., 2010). As SakA accumulation depends on AtfA during conidiation but not during growth (Fig. 5B), it remains to be determined if the SakA pathway regulates the same set of genes during growth and during spore formation.

Many members of the ATF/CREB family of transcription factors bind to DNA consensus ATF/CRE site (TGACGTCA), either as homo- or as heterodimers. In *S. pombe* Atf1 forms heterodimers with Pcr1, a smaller bZIP transcription factor, and Atf1 and Pcr1 have common as well as distinct roles in stress gene regulation (Sanso *et al*., 2008). As our *atfA* probe detected two transcripts with similar but not identical patterns of accumulation, we deleted genes AN8643.3 and AN6849.3, encoding putative bZIP transcription factors, to test the possibility that they could encode the *atfA*-related smaller transcript. Although this was not the case, we found that only the deletion of AN8643.3 resulted in a H_2_O_2_ spore sensitivity similar to the one observed in Δ*atfA* conidia, while spores and mycelia were only moderately sensitive to *t*-BOOH (Fig. S7A). Unexpectedly, CatA accumulation in conidia was not affected in either AN6849.3 or AN8643.3 mutants (Fig. S7B). We propose to name proteins AN8643.3 and AN6849.3 as AtfB and AtfC, respectively, as they show higher similarity to the recently described AtfB and AtfC in *A. oryzae* (Sakamoto *et al*., 2009), AtfB being more similar to *S. pombe* Pcr1. As AtfA and AtfB are both required for spore resistance to oxidative stress, it is possible that they might interact to regulate genes in common, with each transcription factor having also some gene specificity (i.e. AtfA but not AtfB is required for *catA* expression), as it occurs with *S. pombe* Atf1 and Pcr1 (Sanso *et al*., 2008). Using DNA microarrays, Sakamoto *et al*. have recently found that in *A. oryzae* most genes regulated by AtfA are regulated by AtfB, but some genes regulated by AtfA do not require AtfB (Sakamoto *et al*., 2009).

### AtfA and SakA interact to regulate stress signalling

We have not only shown that the MAPK SakA and the transcription factor AtfA are functionally connected but that in fact they physically interact under different conditions of stress and development. Not only null mutants in *sakA* and/or *atfA* show similar phenotypes but also in the absence of *atfA*, SakA fails to accumulate in conidia. Yet, AtfA is localized in the nucleus even in absence of SakA or stress as it occurs in *S. pombe* (Gaits *et al*., 1998; Gaits and Russell, 1999). A lack of *atfA* did not affect *sakA* mRNA (not shown) or SakA protein (Fig. 5B) levels during growing conditions. However, during conidiation AtfA might affect *sakA* mRNA stability. Alternatively, phosphorylated SakA might become unstable if AtfA is not available to interact with. There are positive and negative feedback regulatory loops between Spc1 and Atf1 in *S. pombe*: Spc1 and the RNA-binding protein Csx1 regulate *atf1* mRNA stability (Rodriguez-Gabriel *et al*., 2003). Atf1, in turn, regulates expression of *pyp2*, which encodes a phosphatase that dephosphorylates Spc1. However, a positive feedback like the one found between SakA and AtfA during *A. nidulans* conidiation has not been reported before. It is unlikely that AtfA is the only SakA substrate and AtfA might have functions that are SakA-independent. For example, under nutrient starvation, the accumulation of *catA* mRNA was dependent on SakA and AtfA (involving the SakA–AtfA pathway in carbon and nitrogen starvation signalling). This can explain the lack of CatA activity in Δ*atfA* conidia, but the fact that about 50% of CatA activity is still present in Δ*sakA* conidia (Kawasaki *et al*., 2002; Vargas-Perez *et al*., 2007; this work) suggests that without SakA, AtfA might retain some of its activity, as it has been recently found for Atf1 in *S. pombe* (Reiter *et al*., 2008). The SakA dependence on AtfA during conidiation requires further investigation to fully dissect SakA and AtfA-dependent functions. This is also true during fludioxonil treatment, where a lack of SakA results in about threefold reduction of *atfA* mRNA (Hagiwara *et al*., 2009), making it difficult to define which genes induced by fludioxonil depend on SakA and which might depend only on AtfA.

### The SakA–AtfA pathway plays differential functions in oxidative stress responses during growth and during development

Different mechanisms have been involved in resistance to H_2_O_2_ or *t*-BOOH. Indeed, catalases can only decompose H_2_O_2_ while different members of the peroxiredoxin family can decompose H_2_O_2_ as well as organic hydroperoxides, like *t*-BOOH, with different efficiencies and specificity (Munhoz and Netto, 2004). A recent report showed that Δ*atfA* conidia were sensitive to 50 mM H_2_O_2_ but Δ*atfA* mycelia was resistant to both H_2_O_2_ and *t*-BOOH. This led the authors to conclude that AtfA is important for oxidative stress tolerance in conidia but not in mycelia (Hagiwara *et al*., 2008). In contrast, we found that the SakA–AtfA module plays critical roles in the antioxidant response in both conidia and mycelia and, more importantly, that these roles are not identical. This pathway is required for CatA activity and for spore resistance to both H_2_O_2_ and *t*-BOOH. On the contrary, this pathway is largely dispensable for mycelial resistance to H_2_O_2_ but still needed for *t*-BOOH resistance. This suggests that during conidiation the SakA–AtfA module not only regulates catalase but also peroxiredoxin or other genes involved in *t*-BOOH resistance. During mycelial growth the SakA–AtfA system is not the only one involved in induction of catalase CatB. We reported that other transcription factor, the response regulator SrrA, is critical for induction of catalase CatB by H_2_O_2_ (Vargas-Perez *et al*., 2007). Nevertheless, SakA and AtfA are both needed for mycelial expression of mechanisms dealing with the stress caused by *t*-BOOH. Contrary to this, the Sty1–AtfA1 pathway in *S. pombe* is critical for H_2_O_2_ resistance but plays a minor role in vegetative resistance to *t*-BOOH (Chen *et al*., 2008).

### SAPK phosphorylation, spore dormancy and germination

We found that SAPKs SakA and OS-2 become phosphorylated during the development of the asexual spores in *A. nidulans* and *N. crassa* respectively. It is important to define what type of stimuli trigger the activation of these MAPKs under these conditions and the components involved in the process. In *A. nidulans* SakA becomes activated between 20 and 60 min after conidiation is induced by exposing mycelium to air (Kawasaki *et al*., 2002). However, this phosphorylation is transient and occurs long before the structures that produce conidia (phialides) had been formed. Moreover, SakA and AtfA interaction occurs during conidiophore and conidia development. It is likely that nutritional or other stress signals are produced during spore formation, which trigger SakA phosphorylation in a process that depends on response regulator SskA but not on histidine kinase NikA. Members of the NikA family have been involved in osmotic stress and fludioxonil sensing in different fungi. *A. nidulans* NikA is dispensable for osmostress resistance but it is essential for fludioxonil signalling to response regulators SrrA and SskA (Vargas-Perez *et al*., 2007). In *N. crassa* the MAPK OS-2 is not only regulated by stress signalling, but also by an endogenous circadian rhythm. However, as this also occurs in the absence of spore formation (Vitalini *et al*., 2007), it is possible that the OS-2 phosphorylation we found in conidia results from physiological stress signalling.

In both fungi, phosphorylated SAPKs become dephosphorylated during spore germination. We showed that permanent phosphorylation of SakA, induced by fludioxonil, prevents germ tube formation and nuclear division and that these fludioxonil effects are bypassed by the lack of SakA (caused by deletion of either *sakA* or *atfA*). Thus, it is likely that SAPK phosphorylation in spores is linked to cell cycle control. *S. pombe spc1* was identified as a gene required to respond to environmental stress and to promote the onset of mitosis (Shiozaki and Russell, 1995) and *sakA* expression in *S. pombe* induced a small-cell phenotype consistent with an advancement in the cell cycle (Kawasaki *et al*., 2002). More recently, it has been found that different degrees of Sty1 (Spc1) activation (phosphorylation) determine mitotic commitment decisions during nutrient starvation (Hartmuth and Petersen, 2009) and other stress conditions (Shiozaki, 2009).

The phosphorylation of SakA in conidia and the lack of germination and nuclear division when phosphorylation is maintained are consistent with a SakA role in spore cell-cycle arrest. There are other instances, perhaps related to SakA function, where cell-cycle arrest prevents spore germination in *A. nidulans*. Certainly, the expression of a constitutively active form of a calcineurin-dependent kinase (CaMK) in spores prevents germination and entry into the cell cycle (Dayton *et al*., 1997). We propose that high SAPK phosphorylation in spores is needed for cell-cycle arrest and spore dormancy and that decreased phosphorylation levels are needed to resume growth and mitosis during germination. Taken together, our results indicate that SAPK phosphorylation plays an essential role in fungal spore function and perhaps a more general role in different types of cell dormancy and/or cell-cycle arrest in eukaryotic cells.

## Experimental procedures

### *atfA* cloning and sequencing, deletion of *atfA* and *sakA*, AtfA and SakA tagging

To identify a possible Atf1 orthologue in *A. nidulans*, a public cDNA database (Prade *et al*., 2001) was searched using *S. pombe* Atf1 protein sequence as probe. A partial cDNA sequence was recognized and used to design primers to generate a probe, using genomic DNA as template. This probe was hybridized to a chromosome-specific library (Brody *et al*., 1991). Cosmid W16G04 was identified and used to obtain the genomic sequence deposited before at GenBank (*atfA* gene; Accession No. AY166595). An *atfA* cDNA was obtained using primers ATF9 and ATF10, total mRNA from wild-type mycelia grown for 18 h and SuperScript III One-step RT-PCR Platinum high-fidelity Taq (Invitrogen, Carlsbad, CA). The *atfA* cDNA sequencing was carried out using primers ATF5-10. DNA sequence was obtained by automatic fluorescent dideoxy sequencing in a Perkin-Elmer ABI Prism 310 sequencer. Genomic DNA was used as template to produce an *atfA* gene replacement construct by double joint PCR (Yu *et al*., 2004). The 5′*atfA* fragment was obtained with primers 5′AtfFor and 5′AtfRev+Tail (see Table 1 primers sequence). The 3′*atfA* fragment was amplified with primers 3′AtfFor+Tail and 3′AtfRev. The *Aspergillus fumigatus pyrG* marker was amplified with primers pyrGforward and pyrGreverse, using plasmid PFNO3 as template (Nayak *et al*., 2006). The three fragments were purified, mixed and used in a fusion PCR with primers NestedForward and NestedReverse. The final 6064 bp atfA–AfpyrG–AtfA cassette was purified and used to transform *A. nidulans* strains CFL3 and 11035 by electroporation (Sanchez and Aguirre, 1996; Sanchez *et al*., 1998). A similar strategy was used to delete the *sakA* gene, using primers 5′For-sakA and 5′Rev-sakA for 5′ region; 3′For-sakA and 3′Rev-sakA for the 3′ region; and primers 5′Nest-sakA and 3′Nest-sakA for the fusion product. In this case, the *A. fumigatus riboB* marker was amplified with primers 5Ribo and 6Ribo, using plasmid pAfriboPstE1Skt(ssp1)-37 as template (Nayak *et al*., 2006). The 4773 bp sakA–ribo–sakA cassette was purified as before and used to transform *A. nidulans* strain 11035 by electroporation. Three PCR products were used to generate an AtfA C-terminal GFP construct, according to Yang *et al*. (2004). First, a 5′ fragment upstream the stop codon, including the entire *atfA* ORF, was amplified with primers GSP1atfA and GSP2atfA. Second, a 3′*atfA* fragment was amplified with primers GSp3atfA and GSP4atfA. Third, GFP and *A. fumigatus pyrG* marker were amplified with primers GFP1atfA and GFP2atfA, using plasmid PFNO3 as template (Nayak *et al*., 2006). Purified fragments were mixed and used in a fusion PCR with primers GSP1atfA and GSP4atfA. The 6708 bp atfA–GFP–AfpyrG cassette was used to transform *A. nidulans* strain CFL3 by electroporation. To generate a SakA::GFP construct, 5′ and 3′ fragments were amplified with primers GSP1sakA, GSP2sakA and GSP3sakA, GSPsakA4 respectively. GFP and *A. fumigatus pyrG* marker were amplified with primers GFP1sakA and GFP2sakA. A 5963 bp fusion PCR product obtained with primers 5NestSakA–GFP and 3′Nest-sakA was used to transform strain A1155. The *atfA::gfp* allele was introduced into a Δ*sakA* genetic background by sexual crosses, to generate strain CFL9.

**Table 1:** DNA primers used in this study

Primer	Sequence (5′ to 3′)
5′AtfFor	ATACCGCTACGAATCCAGACCC
5′AtfRev+Tail	AGAGGGTGAAGAGCATTGTTTGAGGCATGGGAAAGATAACATCGGAGG
3′AtfFor+Tail	TCACGCATCAGTGCCTCCTCTCAGACACTTGAAGACACTGTTACTTGC
3′AtfRev	AAGCCTCAAATCTCTATCTACC
5′Nest-atfA	GCCGTATCCCCTAAGCGTTTCC
3′Nest-AtfA	GCCATCATCTATCTTGTTCCAC
5′For-sakA	GTACATCGACGACGGCTG
5′Rev-sakA	CCATGTGATCAAACGAGCCAGTCCGCCATTTTGATCGAG
3′For-sakA	CATGTAACGGTTCTGCAGCGCAATGAATCGCGTGGATGC
3′Rev-sakA	CGAGATCCGAAAAGTCCC
5′Nest-sakA	TGGAGCGGTAAAGCGTCC
3′Nest-sakA	TCAGCAAGCATCCCAAGG
GFP1atfA	CTTATCTGGAACCCGAATCCTCCATACACTGGAGCTGGTGCAGGCGCTGGAGCCGGTGCC
GFP2atfA	TGCGCCAGTTAGTCAAGTGATATTATTCCTGTCTGAGAGGAGGCACTGATGC
GSP1atfA	CGCGCTCTCCCTTCGTTTCTCTATACTG
GSP2atfA	AGTGTATGGAGGATTCGGGTTCCAGATAAG
GSP3atfA	AGGAATAATATCACTTGACTAACTGGCGCA
GSP4atfA	GGCCATACTTACCTTTAAGCCTCAAATC
ATF5	TAACAGTGGTCGTAAGCG
ATF7	GCTTACGACCACTGTTAC
ATF8	AGCATAGCAGGACTCAGG
ATF9	GCCTCTCTATTATTCAGC
ATF10	ACTGGAACTCAACCTGCGC
pyrGforward	GCCTCAAACAATGCTCTTCACC
pyrGreverse	GTCTGAGAGGAGGCACTGATGC
5Ribo	CTGGCTCGTTTGATCACATGG
6Ribo	GCGCTGCAGAACCGTTACATG
CATA1	ATGGCTACTAGTATCACC
CATA2	TTAGAACGCAACCGTCGA
For-sakA	CACCATGGCGGAATTTGTACGTGC
Rev-sakA	TTGGAAACCTTGCTGGTTGAGC
3AtfFor-ribo	CCATGTGATCAAACGAGCCAGATGGGAAAGATAACATCGGAGG
3AtfRev-ribo	CATGTAACGGTTCTGCAGCGCACTTGAAGACACTGTTACTTGC
GSP1sakA	GCCTGCCCAAGTGCGTGCGGTCTATT
GSP2sakA	TTGGAAACCTTGCTGGTTGAGCCCGGCTCC
GFP1sakA	GGAGCCGGGCTCAACCAGCAAGGTTTCCAAGGAGCTGGTGCAGGCGCTGGAGCCGGTGCC
GFP2sakA	AGCGCCGCATCCACGCGATTCATTAGGGCTGTCTGAGAGGAGGCACTGATGC
GSP3sakA	AGCCCTAATGAATCGCGTGGATGCGGCGCT
GSP4sakA	CATGCATAGCCGTTCGCATCTTGAGCCG
5NestSakA–GFP	TCCCCACACACCATTCATTCG
5sakA–YFP	GGGGCGCGCCCATGGCGGAATTTGTACGTGCC
3sakA–YFP	CCTTAATTAAGGATTTTCCGTCACCACAACG
5atfA–YFP	GGGGCGCGCCAATGTCTGCCGCCGTGGCTTCG
3atfA–YFP	CCTTAATTAACCTGATTGCACTGCTAAACAGC

For bimolecular fluorescence complementation experiments (BiFC) SakA and AtfA were tagged with separated halves of YFP, based on reported methodology (Takeshita *et al*., 2008), with the following modifications. To fuse YFP-N to SakA, a 2.9 kb *sakA* PCR product was amplified with primer 5sakA–YFP (which introduces an AscI restriction site before the starting ATG) and primer 3sakA–YFP (which introduces a PacI restriction site 1.5 kb after the stop codon). To fuse YFP-C to AtfA, primers 5atfA–YFP and 3atfA–YFP were used to generate a 3.1 kb PCR product. Both products were cloned into pGEM-T EASY vector (Promega, Madison, WI) yielding plasmids pGEMT-sakA (5954 bp), and pGEMT-atfA (6216 bp) respectively. To generate plasmid PFL-01 (*YFP-N::sakA*), a *sakA* AscI–PacI fragment was used to replace *uncA* in plasmid pNZ-SI39, which contains YFP-N under the regulation of the *alcA* promoter and *A. fumigatus pyroA* as genetic marker. For plasmid PFL-02 (*YFP-C::atfA*) an *atfA* AscI–PacI fragment was used to replace *uncA* in plasmid pNZ-SI40 containing YFP-C regulated by the *alcA* promoter and *N. crassa pyr4* as marker. PFL-01 and PFL-02 were used to co-transform *A. nidulans* strain 11035. Plasmids pNZ-SI39 and pNZ-SI40 were generous gifts from Nadine Zeckert and Reinhard Fischer (Karlsruhe Technical University).

### Microscopy

Samples of conidia or mycelia were fixed in 3.7% formaldehyde, 50 mM Na_2_HPO_4_ (pH 7.0) and 0.2% tween 80 for 30 min. Samples were washed in water and stained for 5 min with 0.1 µg ml^−1^ 4′,6-diamidino-2-phenylindole (DAPI) and preparations were examined under a NIKON Eclipse E600 microscope, to detect DAPI and GFP fluorescence. For live-cell imaging of germlings and young hyphae treated with H_2_O_2_, conidia from SakA::GFP strain were grown on coverslips submerged in liquid minimal medium for 12 h at 37°C and no shaking. Coverslips were transferred to the same medium containing 30 mM H_2_O_2_ for 30 min and GFP fluorescence was observed using a NIKON Eclipse E600 microscope. Cultures on solid media were observed with a confocal microscope Olympus FV1000. For H_2_O_2_ treatment during the BiFC assays, conidia were grown as before on coverslips in liquid 1% glucose minimal medium for 12 h at 37°C and then transferred to 100 mM threonine-minimal medium for 3 h to induced the *alcA* promoter. After this time cultures were treated or not with 30 mM H_2_O_2_ for 30 min and examined using a confocal microscope Olympus FV1000. For cultures observations on solid media, medium contained 100 mM threonine as sole carbon source and pictures were taken using the confocal microscope Olympus FV1000. YFP fluorescence was coloured in yellow for clarity.

### Strains, media and growth conditions

*Aspergillus nidulans* strains used in this work are listed in Table 2. All strains were grown at 37°C in glucose minimal nitrate medium (Hill and Käfer, 2001), plus supplements. Menadione, paraquat and methylglyoxal were filter sterilized and like H_2_O_2_ and *t*-butylhydroperoxide, added to agar medium at ∼50°C before solidification. H_2_O_2_-containing plates were used the day they were prepared or stored at 4°C for no more than 24 h. Since H_2_O_2_ can react with medium components, the actual concentration in plates cannot be estimated. To ensure reproducibility, the same batch of H_2_O_2_-containing medium was used when comparing different strains. Induction of catalase CatB by H_2_O_2_ or paraquat, and in-gel specific catalase activity was carried out as reported (Kawasaki *et al*., 1997; Kawasaki and Aguirre, 2001). The activity of superoxide dismutase SodA was determined using the in-gel nitroblue tetrazolium (NBT) assay (Flohe and Otting, 1984). Total protein was determined by the method of Bradford. To induce sexual development, conidia were plated on top agar at 1 × 10^5^ conidia per plate, incubated at 37°C and cleistothecia were counted as reported (Kawasaki *et al*., 2002).

**Table 2:** *Aspergillus nidulans* strains used in this study

Strains	Genotype	Source
CLK43	*pabaA1, yA2; veA1*	Kawasaki *et al*. (2002)
TOL1	*pabaA1, yA2;*Δ*argB::trpC*Δ*B;*Δ*sakA::argB; trpC801, veA1*	Kawasaki *et al*. (2002)
TFLΔatfA-02	*pabaA1, yA2; pyrG89;*Δ*atfA::AfpyrG; veA1*	This work, CFL3 transformed with PCR construct atfA–AfpyrG–atfA
TRN1	*pabaA1, yA2;*Δ*argB::trpC*Δ*B;*Δ*cata::argB*Δ*B; trpC801, veA1*	Navarro *et al*. (1996)
TLK12	*pabaA1, yA2;*Δ*argB::trpC*Δ*B;*Δ*catB::argB; trpC801, veA1*	Kawasaki *et al*. (1997)
CFL3	*pabaA1, yA2; pyrG89; veA1*	This work, progeny from CLK43 X SRF200
SRF200	*pyrG89; ΔargB::trpC; pyroA4; veA1*	R. Fischer
11035	*pyrG89; pyroA4; riboB2; ΔnkuA::argB; veA1*	M. Hynes; Nayak *et al*. (2006)
TFLΔsakA-03	*pyrG89; pyroA4; riboB2; ΔsakA::AfriboB; ΔnkuA::argB; veA1*	This work, 11035 transformed with PCR construct sakA–AfriboB–sakA
TFL4; TFL5	*pyrG89; pyroA4;riboB2; ΔsakA::AfriboB;*Δ*atfA::AfpyrG; ΔnkuA::argB; veA1*	This work, TFLΔsakA-03 transformed with PCR construct atfA–AfpyrG–atfA
TFL3	*pabaA1, yA2; atfA::GFP::AfpyrG; veA1*	This work, CFL3 transformed withPCR construct atfA–GFP–AfpyrG
CFL9	*pabaA1, yA2; ΔsakA::AfriboB; atfA::GFP::AfpyrG; veA1*	This work, progeny from TFL3 X TFLΔsakA-03
TFLΔatfA-04	*pyrG89; pyroA4; riboB2; ΔatfA::AfriboB; ΔnkuA::argB; veA1*	This work, 11035 transformed with PCR construct atfA–AfriboB–atfA
COSΔsrrA03	*pabaA1, yA2;*Δ*srrA::AfpyrG; veA1*	Vargas-Perez *et al*. (2007)
COSΔsskA02	*pabaA1, yA2,*Δ*sskA::AfriboB; veA1*	Vargas-Perez *et al*. (2007)
COSΔsrrA/ΔsskA02	*pabaA1, yA2;*Δ*sskA::AfriboB; veA1*	Vargas-Perez *et al*. (2007)
CIVΔnikA3	*pabaA1, yA2;*Δ*nikA::AfpyrG; veA1*	Vargas-Perez *et al*. (2007)
A1155	*pyrG89; pyroA4; ΔnkuA::bar; veA1*	Fungal Genetic Stock Center
TFL6	*pyrG89;pyroA4; sakA::GFP::AfpyrG; ΔnkuA::bar; veA1*	This work, A1155 transformed withPCR construct sakA–GFP–AfpyrG
TFL7	*pyrG89; pyroA4; riboB2; alcA::YFP-C::atfA::pyr4; alcA::YFP-N::sakA::pyroA; ΔnkuA::argB; veA1*	This work, 11035 transformed with plasmids PFL-01 and PFL-02

### Immunoblot detection

*Aspergillus nidulans* or *N. crassa* samples were prepared from intact or germinated conidia. For germination, 1 × 10^7^ conidia were used to inoculate 200 ml of liquid cultures and incubated at 37°C and shaking at 250 r.p.m. At indicated time samples were filtered through 0.22 µm membranes (Millipore, Bedford, MA), frozen with liquid nitrogen, grounded in a mortar and immediately resuspended in Laemmli's SDS/DTT sample buffer without the dye and maintained on ice. Samples were further disrupted by vortexing with glass beads and cell debris was removed by centrifugation. Total protein (50 µg) was used for immunoblotting as reported before (Kawasaki *et al*., 2002) using Hog1 (y-215) polyclonal (Santa Cruz Biotechnology, CA) and Phospho-p38 MAP Kinase Antibodies (Cell Signaling Technology, Beverly, MA). Blots were also probed with anti-α-tubulin (Sigma, Steinheim, Germany) antibodies for protein loading control. HRP-conjugated secondary antibodies (Zymed Laboratories, San Francisco, CA) and PIERCE supersignal chemiluminescent substrate (Thermo Scientific, Rockford, IL) were used for detection.

### RNA extraction and Northern blot analysis

Mycelial or conidial samples were frozen in liquid nitrogen and stored at −70°C until used and ground with mortar and pestle under liquid nitrogen. Total RNA was isolated with Trizol (Invitrogen, Carlsbad, CA) according to the manufacturer's instructions. Ten to 15 µg of RNA was separated in a 1% agarose gel containing formaldehyde, transferred to Hybond N membranes (Amersham Biosciences, Piscataway, NJ) and the membrane was hybridized using specific probes.
