# Supplementary material for: Aspergillus nidulans transcription factor AtfA interacts with the MAPK SakA to regulate general stress responses, development and spore functions
Source: Mol Microbiol. 2011 Mar 1;80(2):436–54. doi: 10.1111/j.1365-2958.2011.07581.x (PMC3108070; doi:10.1111/j.1365-2958.2011.07581.x)
Supplement: Supplementary file 1 [file mmi0080-0436-SD1.pdf]

```
A.nidulans AtfA 485 aa       1   -----MSAAVASAVSTTLPSNP-TAHSSPMDAKKNSVKMDN-EASSETKE 43
A.oryzae BAD93190 366 aa     1   -------------------------------------------------- 1
C.purpurea CAD21519 550 aa   1   -----MGTSPTNESKSPDLSSNKTRQDPTPGTKVDSVVKPN--------- 36
S.pombe BAA12194 566 aa      1   MSPSPVNTSTEPASVAAVSNGNATASSTQVPENNQSDSFAPPSNNSQQNQ 50

A.nidulans AtfA 485 aa       44  QKTDGEPQTSLAPPSRPNPSAATDTP-DYFN-SVHNPFALEPNPFEQSFG 91
A.oryzae BAD93190 366 aa     1   -------------------------------------------------- 1
C.purpurea CAD21519 550 aa   37  GASNAEPTKPLAPPPRPHQQPQGNTTSDYFAGQVAGSLSLEPNPFEQSFG 86
S.pombe BAA12194 566 aa      51  QSSTIAPNGGAGSVANANPADQSDGVTPSFVGSLK--LDYEPNPFEHSFG 98

A.nidulans AtfA 485 aa       91  ----------------------GGS-GETPGKSILPPVASITSPALPG--- 116
A.oryzae BAD93190 366 aa     1   --------------------------------------------------- 1
C.purpurea CAD21519 550 aa   86  ----------------------GGGGPETPGGTKLPSVASLTSPSSLL--- 112
S.pombe BAA12194 566 aa      99  STASVGQGNPSLNRNPSLSNIPSGVPPAFARTLLPPVSSIASPDILSGAP 148

A.nidulans AtfA 485 aa       116 ------------TSSAGGAYNWSN-SLRSGPLSPAMLAGPAGGSDYFDS- 152
A.oryzae BAD93190 366 aa     1   ------------------------------------MLAGPTGSSDYFDS- 14
C.purpurea CAD21519 550 aa   112 ------------PGGNSTPFNWGGGSLRTGPLSPAMLSGPAN--DYFSDS 148
S.pombe BAA12194 566 aa      149 GIASPLGYPAWSAFTRGTMHNPLSPAIYDATLRPDYLNNPSDASAAARFS 198

A.nidulans AtfA 485 aa       152 --IGRGFPTPNESSLRTGLTPGGGGSMFPAPSPNSQALLNQLQNGGATPS 200
A.oryzae BAD93190 366 aa     14  --IGRGFPTPNESSLRTGLTPGGGGSMFPAPSPNSQALLQQLQSGGATPS 62
C.purpurea CAD21519 550 aa   149 HHLRGGFPTPNESSLRSGLTPGGSGSMFPAPTPTSQALFSQLASGGATPS 198
S.pombe BAA12194 566 aa      198 --SGTGFTPGVNEPFRSLLTPTGAG--FPAPSPGTANLLGFHTFDSQFPD 244

A.nidulans AtfA 485 aa       201 TIEFHRTALN-----------VKKNGIA-PTSNP---------------T 223
A.oryzae BAD93190 366 aa     63  TIEFHRTALNA----------AKKNALNGPTSNP---------------T 87
C.purpurea CAD21519 550 aa   199 AIDFHRTAINAAAAKREQGMPPRQSQHQQPTQSQQLQHSQQQSHQQHQQH 248
S.pombe BAA12194 566 aa      245 QYRFTPRDGKP----------PVVNGTNGDQSDYFGANAAVHGLCLLSQV 284

A.nidulans AtfA 485 aa       224 GEGDQVPQNITTT--------MDIKPAQP-ATVDFGPHDAADAANGLFML 264
A.oryzae BAD93190 366 aa     88  SDPEQASQNTN----------MDMKPNQP---DPFGHHDAADAANGLFML 124
C.purpurea CAD21519 550 aa   249 QHQQQGAQSATSAPPDITNGVPAVKLEQKQPSGPFDPHDN-DAANGLFML 297
S.pombe BAA12194 566 aa      285 PDQQQKLQQPISSEND-----QAASTTANNLLKQTQQQTFPDSIRPSFTQ 329

A.nidulans AtfA 485 aa       265 AKGGQSTANQFAAVSNQTAIPPQTLQTSEILQDQNAARRQSVNVNG-VAN 313
A.oryzae BAD93190 366 aa     125 AKGGQANPNQFA-VSNQSSIPPQNIQNND--QARDSDRRTS---NG---- 164
C.purpurea CAD21519 550 aa   298 AQGAQNRNGAQAPSHYNTTSAPSHAHPAPISSSQNPNPSPQMSSGKAVSR 347
S.pombe BAA12194 566 aa      330 NTNPQAVTGTMNPQASRTQQQPMYFMGSQQFNGMPSVYGDTVNPADPSLT 379

A.nidulans AtfA 485 aa       314 TREPSGDGSE-QSEQAKP-ARGRGKRNTSTKASSTGN-RRKTDDS-TQGS 359
A.oryzae BAD93190 366 aa     165 GRETSGDVSDVQGEQAKPATKGK-KKNTATKTSGAANNRRKADDAPVKGS 213
C.purpurea CAD21519 550 aa   348 GVSEATNGSG-DSEQAKPLPKGKGKKTAAS----ATNGRRKAEDMPTKAP 392
S.pombe BAA12194 566 aa      380 LRQTTDFSGQNAENGSTNLPQKTSNSDMPTANSMPVKLENGTDYSTSQEP 429

A.nidulans AtfA 485 aa       360 NKRTKLNNGAASTESPSE-GESEEEEQQPAQKKKAGDTKKMTDEEKRKNF 408
A.oryzae BAD93190 366 aa     214 NKKAK--LSSGSTEPPSDAGDSEEEEEQ--KKKSQSDSKKMTDEEKRKNF 259
C.purpurea CAD21519 550 aa   393 PTKKAKGMPETMNGSVNEEDSDDDDDDM--MGEDGNPKVKMTDEEKRKNF 440
S.pombe BAA12194 566 aa      430 SSNANNQSSPTSSINGKASSESANGTSYS-KGSSRRNSKNETDEEKRKSF 478

                                                     bZIP
A.nidulans AtfA 485 aa       409 LERNRVAALKCRQRKKQWLANLQAKVELFTSENDALTTTVTQLREEIVNL 458
A.oryzae BAD93190 366 aa     260 LERNRVAALKCRQRKKQWLANLQAKVELFTSENDALTATVTQLREEIVNL 309
C.purpurea CAD21519 550 aa   441 LERNRVAALKCRQRKKQWLANLQNKVEMYSSENDALTAQITQLREEVVNL 490
S.pombe BAA12194 566 aa      479 LERNRQAALKCRQRKKQWLSNLQAKVEFYGNENEILSAQVSALREEIVSL 528

A.nidulans AtfA 485 aa       459 KTLLLAHKDCPVSQAQGL--------------------IWN-----PNPP 483
A.oryzae BAD93190 366 aa     310 KTLLLAHKDCPVSQAQGLGPLMMNGMSAGF--DPHPYNIPNNMGMQPGAP 357
C.purpurea CAD21519 550 aa   491 KTLLLAHKDCPVTQQQGLHGPYMAQVVEPYNAQMNPYGMAGPIP-NQQQV 539
S.pombe BAA12194 566 aa      529 KTLLIAHKDCPVAKSNXAAVATS---------------VIGSGDLAQRIX 563

A.nidulans AtfA 485 aa       484 YT--------- 485
A.oryzae BAD93190 366 aa     358 IPTQGLRRQ-- 366
C.purpurea CAD21519 550 aa   540 MAGQGVQRRFS 550
S.pombe BAA12194 566 aa      564 LGY-------- 566
```

Fig. 1S. **The *atfA* gene encodes a putative transcription factor of ATF/CREB family.**

The amino acid sequence of *Aspergillus nidulans* AtfA is aligned with *Schizzosaccharomyces pombe* Atf1, *Claviceps purpurea* Cptf1 and *Aspergillus oryzae* AtfB. Secuences are identified by species name, GenBank accession number and protein size. Underlined sequence indicates the amino acids deleted in the Δ*atfA* mutant. The top red bar indicates the DNA binding domain as deduced from Expasy-Prosite database (http:://www.expasy.org/prosite/).

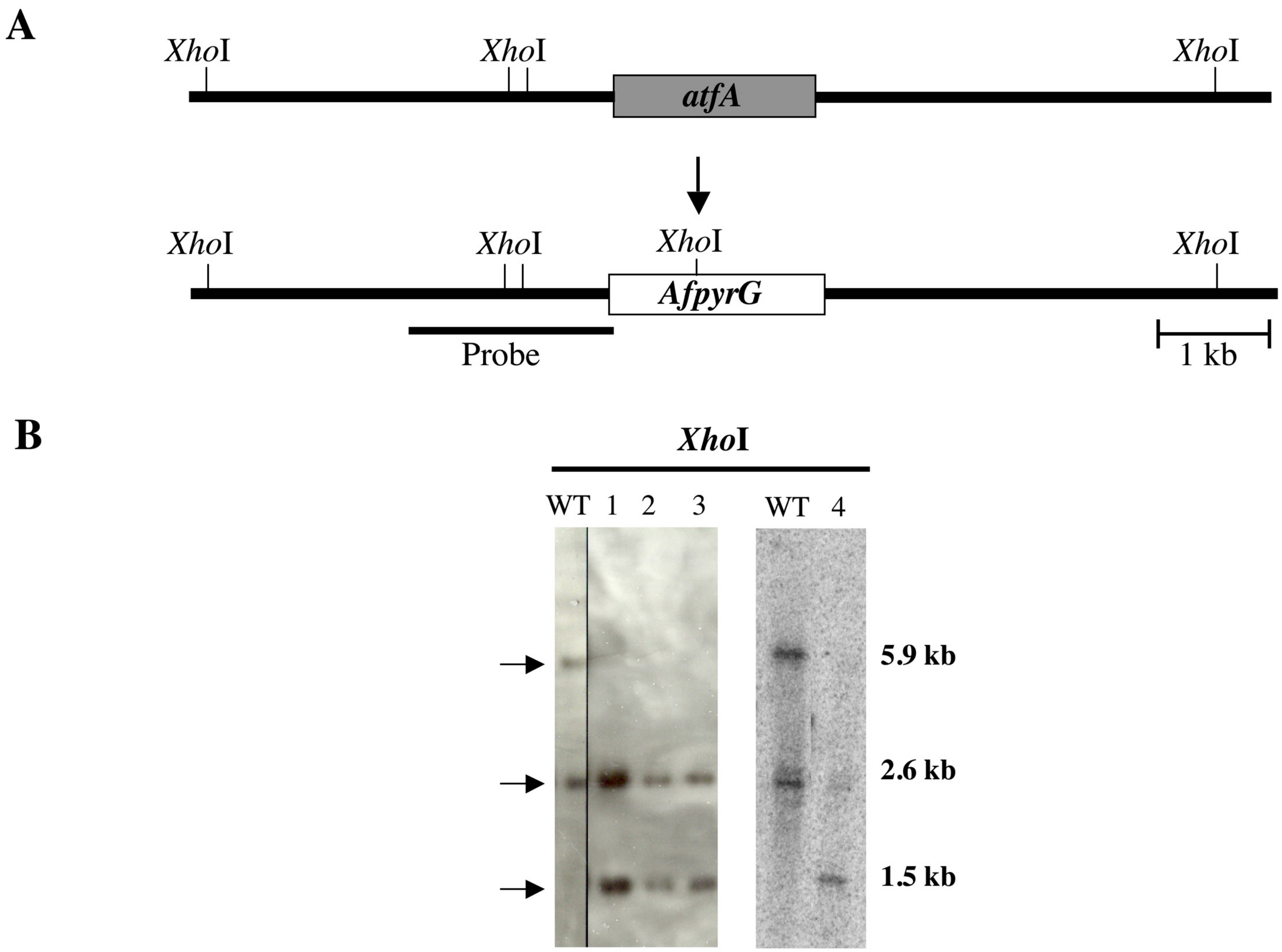

Fig. 2S. **Deletion of the *atfA* gene. (A)** The *atfA* deletion construct containing the *AfpyrG* gene as selective marker was generated by double-joint PCR and used to transform strains CFL3 and 11035. Double recombination results in the replacement of wild type *atfA* by the deletion construct. **(B)** DNA from WT and PyrG$^+$ transformants from CFL3 (1-3) and 11035 (4) strains was digested with *Xho*I and used for Soutern blot analysis with the probe indicated in (A). The wild type *Xho*I pattern correspond to bands of 5.9 and 2.6 kb while Δ*atfA* pattern corresponds to bands of 2.6 and 1.5 kb.

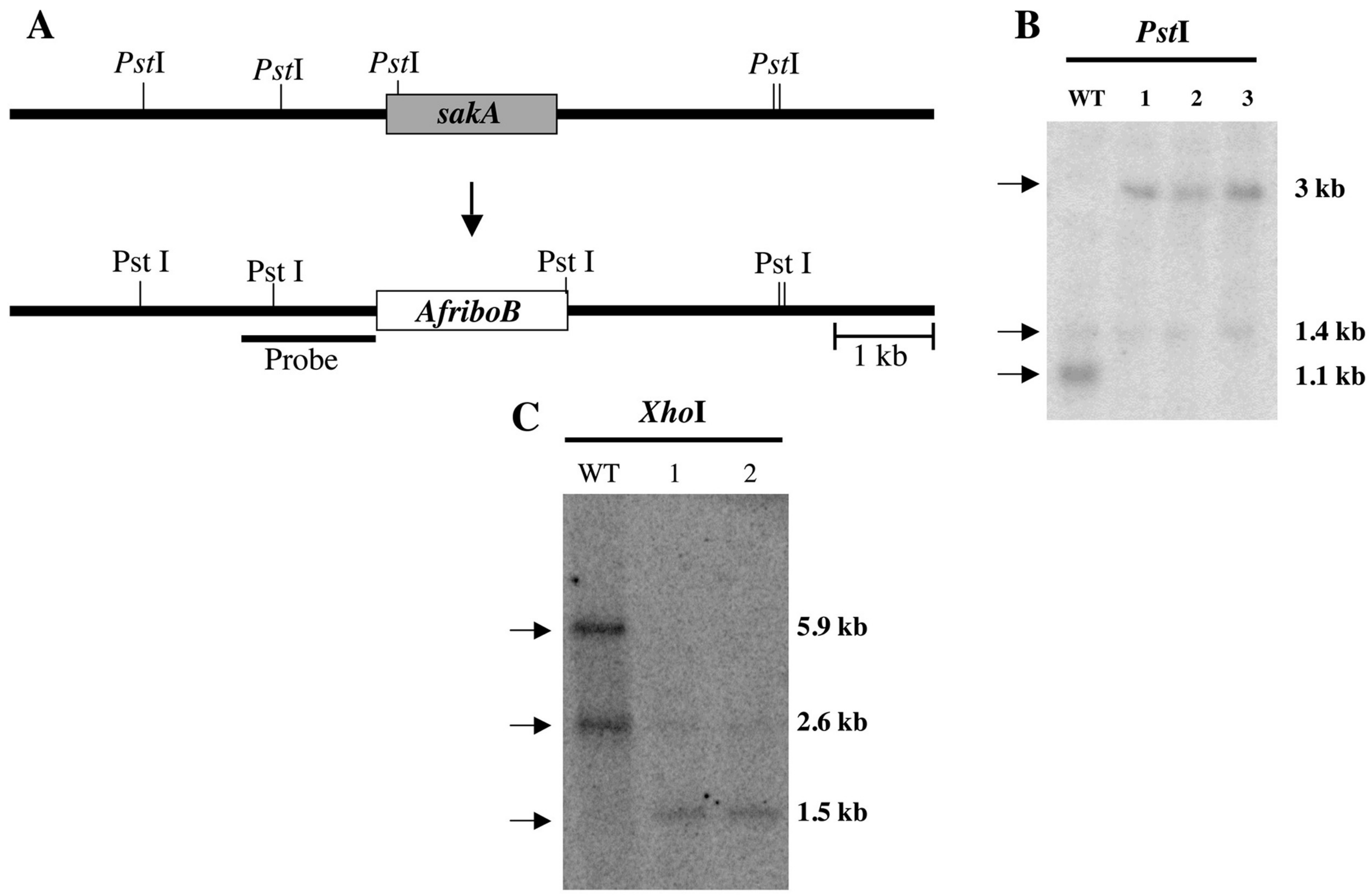

Fig. 3S. **Deletion of the *sakA* gene.** **(A)** A *sakA* deletion construct containing the *AfriboB* gene as selective marker was used to transform strain 11035. **(B)** To verify *sakA* deletion DNA of WT and RiboB+ transformants (1-3) was digested with *Pst*I and used for Southern blot analysis with the indicated probe (A). Wild type *Pst*I pattern correspond to bands of 1.4 and 1.1 kb; Δ*sakA* pattern corresponds to bands of 1.4 and 1.1 kb. **(C)** Δ*sakA* Δ*atfA* double mutants were generated by transformation of Δ*sakA* strain TFLΔsakA-03 with the *atfA-AfpyrG* construct. *atfA* deletion was confirmed in PyrG+ transformants 1 and 2, by Southern blot analysis as in Fig. 2S.

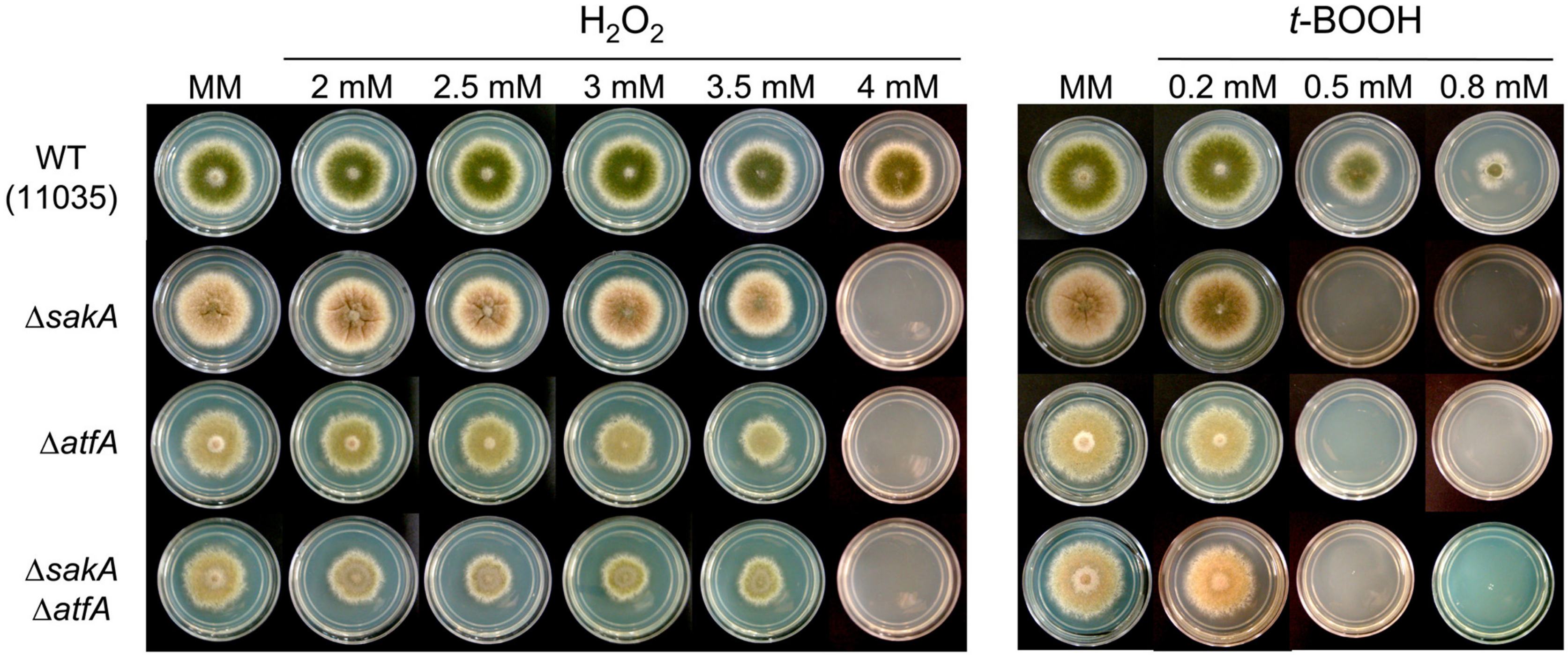

FIG. 4S. **Spores from single and double Δ*sakA* and Δ*atfA* mutants are equally sensitive to H_2O_2 and *t*-BOOH.** Conidia (10^4) from strains 11035 (WT), TFLΔsakA-03 (ΔsakA), TFLΔatfA-04 (ΔatfA) and TFL4 (Δ*sakA* Δ*atfA*) were inoculated on plates containing de indicated concentrations of H_2O_2 or *tert*-butyl hydroperoxide (*t*-BOOH) and incubated for 4 days at 37 °C.

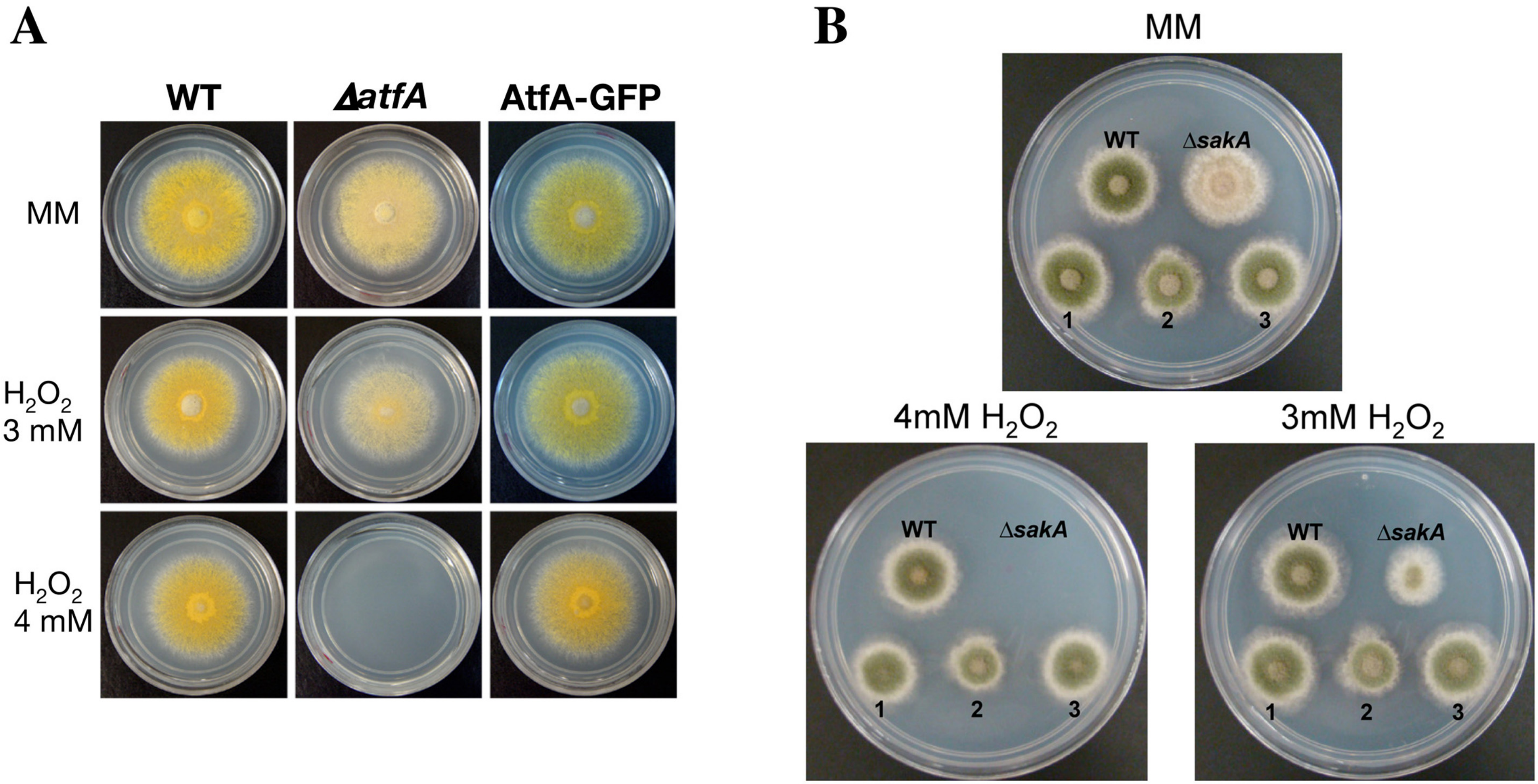

Fig. 5S. **GFP tagging does not interfer with *atfA* or *sakA* function. (A)** Conidiospores (10^4) from strains CLK43 (WT), CFLΔatfA-02 (Δ*atfA*) and TFL3 (*atfA-GFP*) were inoculated on plates containing 3 or 4 mM H_2O_2 and incubated at 37° C for 4 days. **(B)** Conidiospores from strains A1155 (WT), TFLΔsakA-03 (Δ*sakA*) and three *sakA-GFP* transformants were point inoculated on plates containing 3 or 4 mM H_2O_2 and incubated at 37° C for 3 days. Transformant 3 was named TFL6 and selected for further experiments.

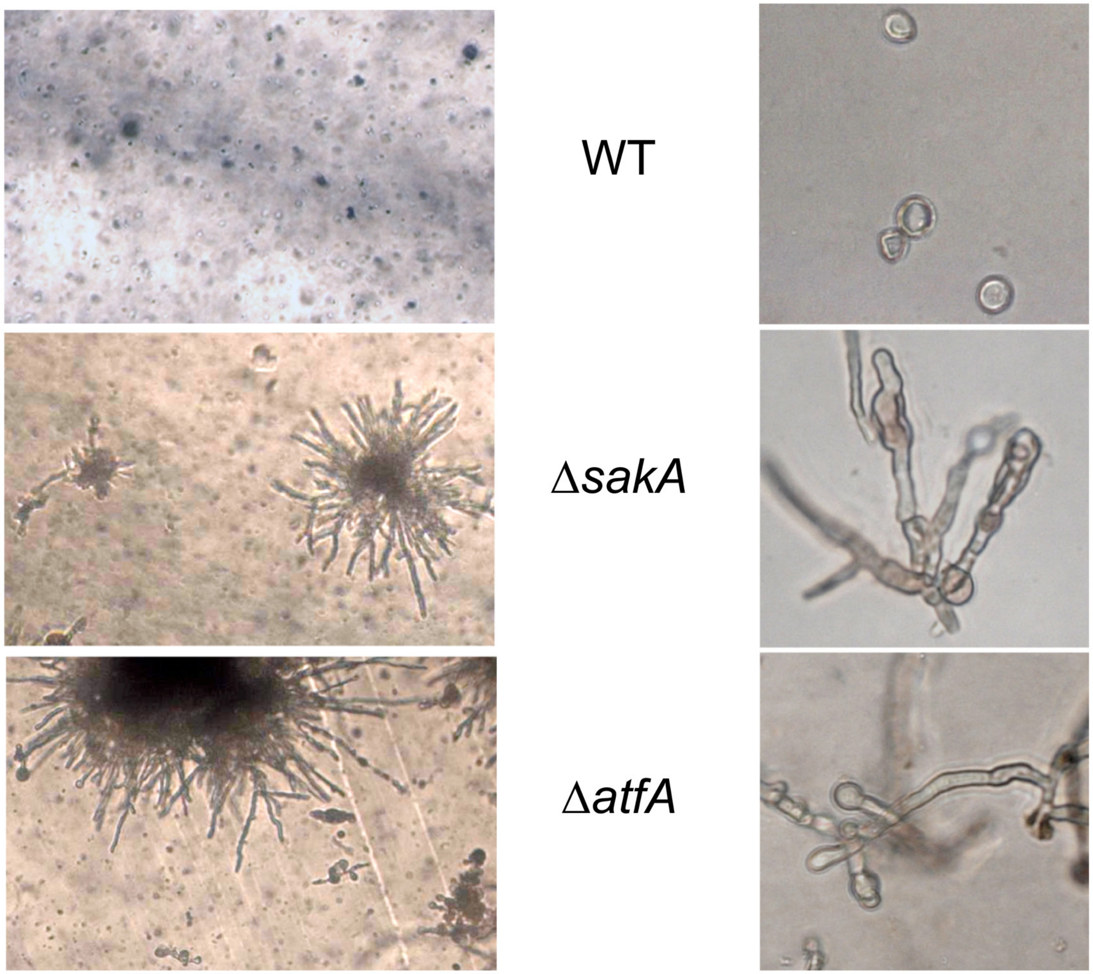

Fig. 6S. **Germination of WT, Δ *sakA* and Δ*atfA* conidia in the presence of fluidoxonil on solid medium**. Conidia from indicated strains were streaked on plates containing minimal solid medium plus 2 μg/ml of fludioxonil and incubated at 37 °C for 4 days. Pictures were taken under the microscope directly from inverted plates (left, 10X objective) or from samples scraped and transferred to a glass slide (right, 40X objective).

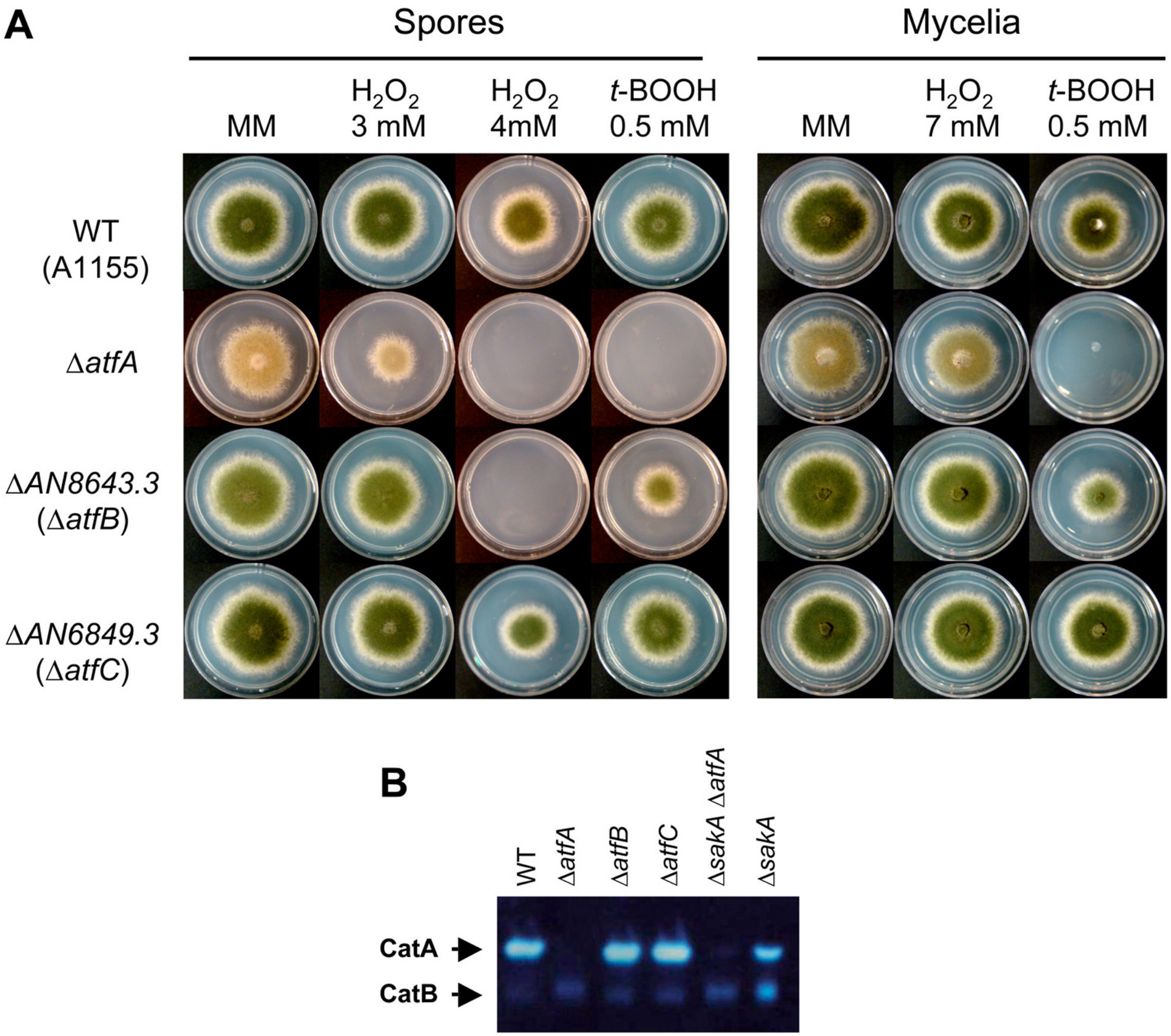

Fig 7S. **Oxidative stress sensitivity and spore catalase activity in mutants lacking putative transcription factors AtfA, AtfB and AtfC**. **(A)** Genes *AN8643.3* (*atfB*) and *AN6849.3* (*atfC*) were deleted in strain A1155, confirmed by Southern blot analysis and tested as in Fig. 1. **(B)** 30 µg of protein extract prepared from spores of the indicated strains were processed as in Fig. 2, to determine catalase activity.
